# Methods for estimating complier average causal effects for cost‐effectiveness analysis

**DOI:** 10.1111/rssa.12294

**Published:** 2017-05-24

**Authors:** K. DiazOrdaz, A. J. Franchini, R. Grieve

**Affiliations:** ^1^ London School of Hygiene and Tropical Medicine UK

**Keywords:** Bivariate outcomes, Cost‐effectiveness, Instrumental variables, Non‐compliance

## Abstract

In randomized controlled trials with treatment non‐compliance, instrumental variable approaches are used to estimate complier average causal effects. We extend these approaches to cost‐effectiveness analyses, where methods need to recognize the correlation between cost and health outcomes. We propose a Bayesian full likelihood approach, which jointly models the effects of random assignment on treatment received and the outcomes, and a three‐stage least squares method, which acknowledges the correlation between the end points and the endogeneity of the treatment received. This investigation is motivated by the REFLUX study, which exemplifies the setting where compliance differs between the randomized controlled trial and routine practice. A simulation is used to compare the methods’ performance. We find that failure to model the correlation between the outcomes and treatment received correctly can result in poor confidence interval coverage and biased estimates. By contrast, Bayesian full likelihood and three‐stage least squares methods provide unbiased estimates with good coverage.

## 1 Introduction

Non‐compliance is a common problem in randomized controlled trials (RCTs), as some participants depart from their randomized treatment, by for example switching from the experimental to the control regimen. An unbiased estimate of the effectiveness of treatment assignment can be obtained by reporting the intention‐to‐treat (ITT) estimand. In the presence of non‐compliance, a complementary estimand of interest is the causal effect of treatment received. Instrumental variable (IV) methods can be used to obtain the complier average causal effect (CACE), as long as random assignment meets the IV criteria for identification (Angrist *et al*., 1996). An established approach to IV estimation is two‐stage least squares, which provides consistent estimates of the CACE when the outcome measure is continuous, and non‐compliance is binary (Baiocchi *et al*., 2014).

Cost‐effectiveness analyses (CEAs) are an important source of evidence for informing clinical decision making and health policy. CEAs commonly report an ITT estimand, i.e. the relative cost‐effectiveness of the intention to receive the intervention (National Institute for Health and Care Excellence, 2013). However, policy makers may require additional estimands, such as the relative cost‐effectiveness for compliers. For example, CEAs of new therapies for end stage cancer are required to estimate the cost‐effectiveness of treatment receipt, recognizing that patients may switch from their randomized allocation following disease progression. Alternative estimates such as the CACE may also be useful when levels of compliance in the RCT differ from those in the target population, or where intervention receipt, rather than the intention to receive the intervention, is the principal cost driver. Methods for obtaining the CACE for univariate survival outcomes have been exemplified before (Latimer *et al*., 2014), but approaches for obtaining estimates that adequately adjust for non‐adherence in CEAs more generally have received little attention. This has been recently identified as a key area where methodological development is needed (Hughes *et al*., 2016).

The context of trial‐based CEA highlights an important complexity that arises with multivariate outcomes more widely, in that, to provide accurate measures of the uncertainty surrounding a composite measure of interest, e.g. the incremental net monetary benefit INB, it is necessary to recognize the correlation between the end points, in this case, cost and health outcomes (Willan *et al*., 2003; Willan 2006). Indeed, when faced with non‐compliance, and the requirement to estimate a causal effect of treatment on cost‐effectiveness end points, some CEAs resort to per‐protocol analyses (Brilleman *et al*., 2015), which exclude participants who deviate from treatment. As non‐compliance is likely to be associated with prognostic variables, only some of which are observed, per‐protocol analyses are liable to provide biased estimates of the causal effect of the treatment received.

This paper develops novel methods for estimating the CACE in CEAs that use data from RCTs with non‐compliance. First, we propose the use of the three‐stage least squares method (Zellner and Theil, 1962), which enables the estimation of a system of simultaneous equations with endogenous regressors. Next, we consider a bivariate version of the ‘unadjusted Bayesian’ models that have previously been proposed for Mendelian randomization (Burgess and Thompson, 2012), which simultaneously estimate the expected treatment received as a function of random allocation, and the mean outcomes as a linear function of the expected treatment received. Finally, we develop a Bayesian full likelihood (BFL) approach, whereby the outcome variables and the treatment received are jointly modelled as dependent on the random assignment. This is an extension to the multivariate case of what is known in the econometrics literature as the IV unrestricted reduced form (Kleibergen and Zivot, 2003).

The aim of this paper is to present and compare these alternative approaches. The problem is illustrated in Section 2 with the REFLUX study, which was a multicentre RCT and CEA that contrasts laparoscopic surgery with medical management for patients with gastro‐oesophageal reflux disease. Section 3 introduces the assumptions and methods for estimating CACEs. Section 4 presents a simulation study that was used to assess the performance of the alternative approaches, which are then applied to the case‐study in Section 5. We conclude with a discussion (Section 6), where we consider the findings from this study in the context of related research.

## 2 Motivating example: cost‐effectiveness analysis of the REFLUX study

The REFLUX study was a UK multicentre RCT with a parallel design, in which patients with moderately severe gastro‐oesophageal reflux disease were randomly assigned to medical management or laparoscopic surgery (Grant *et al*., 2008, 2013).

The RCT randomized 357 participants (178 surgical; 179 medical) from 21 UK centres. An observational preference‐based study was conducted alongside it, which involved 453 preference participants (261 surgical; 192 medical).

For the CEA within the trial, individual resource use (costs in pounds sterling) and health‐related quality of life (HRQOL), measured by using the EuroQol five dimensions questionnaire EQ5D (three levels), were recorded annually for up to 5 years. The HRQOL data were used to adjust life‐years and present quality‐adjusted life‐years (QALYs) over the follow‐up period (Grant *et al*., 2013). (There was no administrative censoring.) As is typical, the costs were right skewed. Table 1 reports the main characteristics of the data set.

**Table 1:** REFLUX study: descriptive statistics^a^

*Statistic*	*Medical*	*Laparoscopic*
	*management*	*surgery*
*N* assigned	179	178
*N* (%) switched	10 (8.3)	67 (28.4)
*N* (%) missing costs	83 (46)	83 (47)
Mean (standard deviation)	1258 (1687)	2971 (1828)
observed cost (£)		
*N* (%) missing QALYs	91 (51)	94 (53)
Mean (standard deviation)	3.52 (0.99)	3.74 (0.90)
observed QALYs		
*Baseline variables*		
*N* (%) missing EQ5D_0_	6 (3)	7 (4)
Mean (standard deviation)	0.72 (0.25)	0.71 (0.26)
observed EQ5D_0_		
Correlation between costs and	−0.42	−0.07
QALYs		
Correlation of costs and QALYs	−0.36	−0.18
by treatment received		

a The follow‐up period is 5 years, and treatment switches are defined within the first year post randomization.

The original CEA estimated the linear additive treatment effect on mean costs and health outcomes (QALYs). The primary analysis used a system of seemingly unrelated regression (SUR) equations (Zellner, 1962; Willan *et al*., 2004), adjusting for baseline HRQOL EQ5D summary score (denoted by EQ5D_0_). The SUR equations can be written for cost *Y*_1*i*_ and QALYs *Y*_2*i*_, as follows:

(1) $$\begin{array}{cc} & Y_{1i}=\beta_{0,\;1}+\beta_{1,\;1}\;{\text{treat}}_{i}+\beta_{1,\;2}{{\text{EQ5D}}_{0}}_{i}+\epsilon_{1i},\\ & Y_{2i}=\beta_{0,\;2}+\beta_{1,\;2}\;{\text{treat}}_{i}+\beta_{2,\;2}{{\text{EQ5D}}_{0}}_{i}+\epsilon_{2i} \end{array}$$

where *β*_1,1_ and *β*_1,2_ represent the incremental costs and QALYs respectively. The error terms are required to satisfy *E*[*ε*_1*i*_]=*E*[*ε*_2*i*_]=0, $E\left[\epsilon_{ki}\epsilon_{k^{\prime }i}\right]=\sigma_{kk^{\prime }}$ and $E[\epsilon_{ki}\epsilon_{k^{\prime }j}]=0$, for $k,k^{\prime }\in \left\{1,2\right\}$, and for *i*≠*j*. Rather than assuming that the errors are drawn from a bivariate normal distribution, estimation is usually done by the feasible generalized least squares (FGLS) method. (If we are willing to assume that the errors are bivariate normal, estimation can proceed by maximum likelihood.) This is a two‐step method where, in the first step, we run ordinary least squares estimation for equation (1). In the second step, residuals from the first step are used as estimates of the elements $\sigma_{kk^{\prime }}$ of the covariance matrix, and this estimated covariance structure is then used to re‐estimate the coefficients in equation (1) (Zellner, 1962; Zellner and Huang, 1962).

In addition to reporting incremental costs and QALYs, CEAs often report the incremental cost‐effectiveness ratio (ICER), which is defined as the ratio of the incremental costs per incremental QALY, and the incremental net benefit INB, defined as INB(*λ*)=*λβ*_1,2_−*β*_1,1_, where *λ* represents the decision makers’ *willingness to pay* for a 1‐unit gain in health outcome. Thus the new treatment is cost effective if INB>0. For a given *λ*, the standard error of INB can be estimated from the estimated increments ${\hat{\beta }}_{1,\;1}$ and ${\hat{\beta }}_{1,\;2}$, together with their standard errors and their correlation following the usual rules for the variance of a linear combination of two random variables. The willingness to pay *λ* generally lies in a range, so it is common to compute the estimated value of INB for various values of *λ*. In the REFLUX study, the reported INB was calculated by using *λ*=£30 000, which is within the range of cost‐effectiveness thresholds that are used by the UK National Institute for Health and Care Excellence (National Institute for Health and Care Excellence, 2013).

The original ITT analysis concluded that, compared with medical management, the arm that was assigned to surgery had a large gain in average QALYs, at a small additional cost, and was relatively cost effective with a positive mean INB, albeit with 95% confidence intervals (CIs) that included zero. However, these ITT results cannot be interpreted as a causal effect of the treatment, since, within 1 year of randomization, 47 of those randomized to surgery switched and received medical management, whereas, in the medical treatment arm, 10 received surgery. The reported reasons for not having the allocated surgery were that, in the opinion of the surgeon or the patient, the symptoms were not ‘sufficiently severe’ or the patient was judged unfit for surgery (e.g. overweight). The preference‐based observational study conducted alongside the RCT reported that, in routine clinical practice, the corresponding proportion who switched from an intention to have surgery and received medical management was relatively low (4%), with a further 2% switching from control to intervention (Grant *et al*., 2013). Since the percentage of patients who switched in the RCT was higher than in the target population and the costs of the receipt of surgery are relatively large, there was interest in reporting a causal estimate of the intervention. Thus, the original study also reported a per‐protocol analysis on complete cases, adjusted for baseline EQ5D_0_, which resulted in an ICER of £7263 per additional QALY (Grant *et al*., 2013). This is not an unbiased estimate of the causal treatment effect, so in Section 5, we reanalyse the REFLUX data set to obtain a CACE of the cost‐effectiveness outcomes, recognizing the joint distribution of costs and QALYs, using the methods that are described in the next section.

## 3 Complier average causal effects with bivariate outcomes

We begin by defining more formally our estimands and assumptions. Let *Y*_1*i*_ and *Y*_2*i*_ be the continuous bivariate outcomes, and *Z*_*i*_ and *D*_*i*_ the binary random treatment allocation and treatment received respectively, corresponding to the *i*th individual. The bivariate end points *Y*_1*i*_ and *Y*_2*i*_ belong to the same individual *i* and thus are correlated. We assume that there is an unobserved confounder *U*, which is associated with the treatment received and either or both of the outcomes. From now on, we shall assume that the *stable unit treatment value assumption* holds: the potential outcomes of the *i*th individual are unrelated to the treatment status of all other individuals (known as *no interference*) and that, for those who actually received treatment level *z*, their observed outcome is the potential outcome corresponding to that level of treatment.

Under the stable unit treatment value assumption, we can write the potential treatment that is received by the *i*th subject under the random assignment at level $z_{i}\in \left\{0,1\right\}$ as *D*_*i*_(*z*_*i*_). Similarly, *Y*_*li*_(*z*_*i*_,*d*_*i*_) with *l* ∈ {1,2} denotes the corresponding potential outcome for end point *l*, if the *i*th subject were allocated to level *z*_*i*_ of the treatment and received level *d*_*i*_. There are four potential outcomes. Since each subject is randomized to one level of treatment, only one of the potential outcomes per end point *l* is observed, i.e. *Y*_*li*_=*Y*_*li*_{*z*_*i*_,*D*_*i*_(*z*_*i*_)}=*Y*_*i*_(*z*_*i*_).

The CACE for outcome *l* can now be defined as

(2) $$\theta_{l}=E\left[\left\{Y_{li}\left(1\right)-Y_{li}\left(0\right)\right\}|\left\{D_{i}\left(1\right)-D_{i}\left(0\right)=1\right\}\right].$$

In addition to the stable unit treatment value assumption, the following assumptions are sufficient for identification of the CACE (Angrist *et al*., 1996).

- *Ignorability of the treatment assignment*:* Z*_*i*_ is independent of unmeasured confounders (conditional on measured covariates) and the potential outcomes $Z_{i}⫫U_{i},D_{i}\left(0\right),D_{i}\left(1\right),Y_{i}\left(0\right),Y_{i}\left(1\right).$
- *The random assignment predicts treatment received*: Pr{*D*_*i*_(1)=1}≠Pr{*D*_*i*_(0)=1}.
- *Exclusion restriction*: the effect of *Z* on *Y*_*l*_ must be via an effect of *Z* on *D*;* Z* cannot affect *Y*_*l*_ directly.
- *Monotonicity*:* D*_*i*_(1)⩾*D*_*i*_(0).

The CACE can now be identified from equation (2) without any further assumptions about the unobserved confounder; in fact, *U* can be an effect modifier of the relationship of *D* and *Y* (Didelez *et al*., 2010).

In the REFLUX study, the assumptions concerning the random assignment, assumptions (a) and (b), are justified by design. The exclusion restriction assumption seems plausible for the costs, since the costs of surgery are incurred only if the patient actually has the procedure. We argue that it is also plausible that it holds for QALYs, as the participants did not seem to have a preference for either treatment, thus making the psychological effects of knowing to which treatment one has been allocated minimal. The monotonicity assumption rules out the presence of defiers. It seems fair to assume that there are no participants who would refuse the REFLUX surgery when randomized to it, but who would receive surgery when randomized to receive medical management. Equation (2) implicitly assumes that receiving the intervention has the same average effect in the linear scale, regardless of the level of *Z* and *U*. This average is, however, across different ‘versions’ of the intervention, as the trial protocol did not prescribe a single surgical procedure, but allowed for the surgeon to choose their preferred laparoscopy method, as would be so in routine clinical practice.

Since random allocation *Z* satisfies assumptions (a)–(c), we say that it is an instrument (or IV) for *D*. For a binary instrument, the simplest method of estimation of equation (2) in the IV framework is the Wald estimator (Angrist *et al*., 1996):

$${\hat{\theta }}_{l,\;\mathrm{IV}}=\frac{E(Y|Z=1)-E(Y|Z=0)}{E(D|Z=1)-E(D|Z=0)}.$$

Typically, estimation of these conditional expectations proceeds via an approach known as two‐stage least squares. The first stage fits a linear regression to treatment received on treatment assigned. Then, in a second stage, a regression model for the outcome on the predicted treatment received is fitted:

(3) $$\begin{array}{cc} D_{i}=\; & \alpha_{0}+\alpha_{1}Z_{i}+\omega_{1i},\\ Y_{li}=\; & \beta_{0}+\beta_{\mathrm{IV}}{\hat{D}}_{i}+\omega_{2i} \end{array}$$

where ${\hat{\beta }}_{\mathrm{IV}}$ is an estimator for *θ*_*l*_. Covariates can be used, by including them in both stages of the model. To obtain the correct standard errors for the two‐stage least squares estimator, it is necessary to take into account the uncertainty about the first‐stage estimates. The asymptotic standard error for the two‐stage least squares CACE is given in Imbens and Angrist (1994) and implemented in commonly used software packages.

Ordinary least squares estimation produces first‐stage residuals *ω*_1*i*_ that are uncorrelated with the instrument, and this is sufficient to guarantee that the two‐stage least squares estimator is consistent for the CACE (Angrist and Pischke, 2008). Therefore, we restrict our attention here to models where the first‐stage equation is linear, even though the treatment received is binary. (Non‐linear versions of two‐stage least squares exist. See for example Clarke and Windmeijer (2012) for an excellent review of methods for binary outcomes.)

A key issue for settings such as CEA, where there is interest in estimating the CACE for bivariate outcomes, is that two‐stage least squares as implemented in most software packages can only be readily applied to univariate outcomes. Ignoring the correlation between the two end points is a concern for obtaining standard errors of composite measures of the outcomes, e.g. INB, as this requires accurate estimates of the covariance between the outcomes of interest (e.g. costs and QALYs).

A simple way to address this problem would be to apply two‐stage least squares directly to the composite measure, i.e. a net benefit two‐stage regression approach (Hoch *et al*., 2006). However, it is known that net benefit regression is very sensitive to outliers, and to distributional assumptions (Willan *et al*., 2004), and has been recently shown to perform poorly when these assumptions are thought to be violated (Mantopoulos *et al*., 2016). Moreover, such net benefit regression is restrictive, in that it does not allow separate covariate adjustment for each of the component outcomes (e.g. baseline HRQOL, for the QALYs as opposed to the costs). In addition, this simple approach would not be valid for estimating the ICER, which is a non‐linear function of the incremental costs and QALYs. For these reasons, we do not consider this approach further. Rather, we present here three flexible strategies for estimating a CACE of the QALYs and the costs, jointly. The first approach combines SUR equations (equation (1)) and two‐stage least squares (equation (3)) to obtain CACEs for both outcomes accounting for their correlation. This simple approach is known in the econometrics literature as three‐stage least squares.

### 3.1 Three‐stage least squares

Three‐stage least squares was developed for SUR systems with *endogenous* regressors, i.e. any explanatory variables which are correlated with the error term in equations (1) (Zellner and Theil, 1962). All the parameters appearing in the system are estimated jointly, in three stages. The first two stages are as for two‐stage least squares, but with the second stage applied to each of the outcomes: first stage,

$$D_{i}=\alpha_{0}+\alpha_{1}Z_{i}+e_{0i};$$

second stage,

(4) $$Y_{1i}=\beta_{01}+\beta_{\mathrm{IV},\;1}\hat{D_{i}}+e_{1i},$$

(5) $$Y_{2i}=\beta_{02}+\beta_{\mathrm{IV},\;2}\hat{D_{i}}+e_{2i}.$$

As with two‐stage least squares, the models can be extended to include baseline covariates. The third stage is the same step used on an SUR with exogenous regressors (equation (1)) for estimating the covariance matrix of the error terms from the two equations (4) and (5). Thus, because *Z* satisfies assumptions (a)–(c), *Z* is independent of the residuals at the first and second stage, i.e. $Z⫫e_{0i}$, $Z⫫e_{1i}$ and $Z⫫e_{2i}$. Then, the three‐stage least squares procedure enables us to obtain the covariance matrix between the residuals *e*_1*i*_ and *e*_2*i*_. As with SURs, the three‐stage least squares approach does not require any distributional assumptions to be made, as estimation can be done by FGLS, and it is robust to heteroscedasticity of the errors in the linear models for the outcomes (Greene, 2002). We note that the three‐stage least squares estimator based on FGLS is consistent only if the error terms in each equation of the system and the instrument are independent, which is likely to hold here, as we are dealing with a randomized instrument. In settings where this condition is not satisfied, other estimation approaches such as generalized methods of moments warrant consideration (Schmidt, 1990). In the just‐identified case, i.e. when there are as many endogenous regressors as there are instruments, classical theory about three‐stage least squares estimators shows that the generalized method of moments and the FGLS estimators coincide (Greene, 2002). As the three‐stage least squares method uses an estimated variance–covariance matrix, it is only asymptotically efficient (Greene, 2002).

### 3.2 Naive Bayesian estimators

Bayesian models have a natural appeal for CEAs, as they afford us the flexibility to estimate bivariate models on the expectations of the two outcomes by using different distributions, as proposed by Nixon and Thompson (2005). These models are often specified by writing a marginal model for one of the outcomes, e.g. the costs *Y*_1_, and then a model for *Y*_2_, conditional on *Y*_1_.

For simplicity of exposition, we begin by assuming normality for both outcomes and no adjustment for covariates. We have a marginal model for *Y*_1_ and a model for *Y*_2_ conditional on *Y*_1_ (Nixon and Thompson, 2005):

(6) $$Y_{1i}∼N\left(\mu_{1i},\sigma_{1}^{2}\right)\;\mu_{1i}=\beta_{0,\;1}+\beta_{1,\;1}{\text{treat}}_{i},$$

(7) $$Y_{2i}|Y_{1i}∼N\left\{\mu_{2i},\sigma_{2}^{2}\left(1-\rho^{2}\right)\right\}\;\mu_{2i}=\beta_{0,\;2}+\beta_{1,\;2}{\text{treat}}_{i}+\beta_{2,\;2}\left(y_{1i}-\mu_{1i}\right),$$

where *ρ* is the correlation between the outcomes. The linear relationship between the two outcomes is represented by *β*_2,2_=*ρσ*_2_/*σ*_1_.

Because of the non‐compliance, to obtain a causal estimate of treatment, we need to add a linear model for the treatment received, dependent on randomization *Z*_*i*_, similar to the first equation of two‐stage least squares. Formally, this model (called uBN, for unadjusted Bayesian normal) can be written with three equations as follows:

(8) $$\left.\begin{array}{cc} & D_{i}∼N\left(\mu_{0i},\sigma_{0}^{2}\right)\;\mu_{0i}=\beta_{0,\;0}+\beta_{1,\;0}Z_{i},\\ & Y_{1i}∼N\left(\mu_{1i},\sigma_{1}^{2}\right)\;\mu_{1i}=\beta_{0,\;1}+\beta_{1,\;1}\mu_{0i},\\ & Y_{2i}|Y_{1i}∼N\left\{\mu_{2i},\sigma_{2}^{2}\left(1-\rho^{2}\right)\right\}\;\mu_{2i}=\beta_{0,\;2}+\beta_{1,\;2}\mu_{0i}+\beta_{2,\;2}\left(y_{1i}-\mu_{1i}\right). \end{array}\right\}$$

This model is a bivariate version of the ‘unadjusted Bayesian’ method that has previously been proposed for Mendelian randomization (Burgess and Thompson, 2012). It is called unadjusted, because the variance structure of the outcomes is assumed to be independent of the treatment received. The causal treatment effect for outcome *Y*_*l*_, with *l* ∈ {1,2}, is represented by *β*_1,*l*_ in equations (8). We use Fisher's *z*‐transform of *ρ*, i.e.

$$z=\frac{1}{2}\text{log}\;\;\left(\frac{1+\rho }{1-\rho }\right)\;\;,$$

for which we assume a vague normal prior, i.e. *z*∼*N*(0,10^2^). We also use vague multivariate normal priors for the regression coefficient (with a precision of 0.01). For standard deviations, we use *σ*_*j*_∼Unif(0,10), for *j* ∈ {0,1,2}. This is similar to the priors that were used in Lancaster (2004) and are independent of the regression coefficient of treatment received on treatment allocation *β*_1,0_.

Cost data are notoriously right skewed, and gamma distributions are often used to model them. Thus, we can relax the normality assumption of equation (8) and model *Y*_1_ (i.e. cost) with a gamma distribution, and treatment received (binary) with a logistic regression. The health outcomes *Y*_2_ are still modelled with a normal distribution, as is customary. Because we are using a non‐linear model for the treatment received, we use the predicted raw residuals from this model as extra regressors in the outcome models, similar to the two‐stage residual inclusion estimator (Terza *et al*., 2008). We model *Y*_1_ by its marginal distribution (gamma) and *Y*_2_ by a conditional normal distribution, given *Y*_1_ (Nixon and Thompson, 2005). We call this model uBGN (unadjusted Bayesian gamma–normal) and write it as follows:

(9) $$\left.\begin{array}{cc} & \text{logit}\left(\pi_{i}\right)=\alpha_{0}+\alpha_{1}Z_{i},\\ & D_{i}∼\text{Bern}\left(\pi_{i}\right),\\ & r_{i}=D_{i}-\pi_{i},\\ & Y_{1i}∼\text{gamma}\left(\nu_{1i},\kappa_{1}\right),\\ & Y_{2i}|Y_{1i}∼N\left\{\mu_{2i},\sigma_{2}^{2}\left(1-\rho^{2}\right)\right\},\\ & \mu_{1i}=\beta_{0,\;1}+\beta_{1,\;1}D_{i}+\beta_{1,\;r}r_{i},\\ & \mu_{2i}=\beta_{0,\;2}+\beta_{1,\;2}D_{i}+\beta_{2,\;r}r_{i}+\beta_{2,\;2}\left(y_{1i}-\mu_{1i}\right), \end{array}\right\}$$

where *μ*_1_=*ν*_1_/*κ*_1_ is the mean of the gamma‐distributed costs, with shape *ν*_1_ and rate *κ*_1_. Again, we express *β*_2,2_=*ρσ*_2_/*σ*_1_ and assume a vague normal prior on Fisher's *z*‐transform of *ρ*,* z*∼*N*(0,10^2^). The prior distribution for *ν*_1_ is gamma(0.01,0.01). We also assume a gamma prior for the intercept term of the cost equation, *β*_0,1_∼gamma(0.01,0.01). All the other regression parameters have the same priors as those used in model uBN.

The models that were introduced in this section, uBN and uBGN, are estimated in one stage, enabling feedback between the regression equations and the propagation of uncertainty. However, these ‘unadjusted’ methods ignore the correlation between the outcomes and the treatment received. This misspecification of the covariance structure may result in biases in the causal effect, which are likely to be more important at higher levels of non‐compliance.

### 3.3 Bayesian simultaneous equations

We now introduce an approach that models the covariance between treatment received and outcomes appropriately, using a system of simultaneous equations. This can be done via full or limited information maximum likelihood, or by using Markov chain Monte Carlo sampling to estimate the parameters in the model simultaneously allowing for proper Bayesian feedback and propagation of uncertainty. Here, we propose a Bayesian approach which is an extension of the methods that were presented in Burgess and Thompson (2012), Kleibergen and Zivot (2003) and Lancaster (2004).

This method treats the endogenous variable *D* and the cost‐effectiveness outcomes as covariant and estimates the effect of treatment allocation as follows. Let (*D*_*i*_,*Y*_1*i*_,*Y*_2*i*_)^T^ be the transpose of the vector of outcomes, which now includes treatment received, as well as the bivariate end points of interest. We treat all three variables as multivariate normally distributed, so that

(10) $$\begin{array}{c} \left(\begin{array}{c} D_{i}\\ Y_{1i}\\ Y_{2i} \end{array}\right)∼N\left\{\left(\begin{array}{c} \mu_{0i}\\ \mu_{1i}\\ \mu_{2i} \end{array}\right)\;\;,\Sigma =\left(\begin{array}{ccc} \sigma_{0}^{2} & s_{01} & s_{02}\\ s_{01} & \sigma_{1}^{2} & s_{12}\\ s_{02} & s_{12} & \sigma_{2}^{2} \end{array}\right)\right\} \end{array},\begin{array}{c} \mu_{0i}=\beta_{0,\;0}+\beta_{1,\;0}Z_{i},\\ \mu_{1i}=\beta_{0,\;1}+\beta_{1,\;1}\beta_{1,\;0}Z_{i},\\ \mu_{2i}=\beta_{0,\;2}+\beta_{1,\;2}\beta_{1,\;0}Z_{i} \end{array}$$

where *s*_*ij*_=cov(*Y*_*i*_,*Y*_*j*_), and the causal treatment effect estimates are *β*_1,1_ and *β*_1,2_. For the implementation, we use vague normal priors for the regression coefficients, i.e. *β*_*m*,*j*_∼*N*(0,10^2^), for *j* ∈ {0,1,2}, *m* ∈ {0,1}, and a Wishart prior for the inverse of Σ (Gelman and Hill, 2006).

## 4 Simulation study

We now use a factorial simulation study to assess the finite sample performance of the alternative methods. The first factor is the proportion of participants who do not comply with the experimental regime, when assigned to it, expressed as a percentage of the total (one‐sided non‐compliance). Bias is expected to increase with increasing levels of non‐compliance. A systematic review (Dodd *et al*., 2012) found that the percentage of non‐compliance was less than 30% in two‐thirds of published RCTs, but greater than 50% in a tenth of studies. Here, two levels of non‐compliance are chosen: 30% and 70%. As costs are typically skewed, three different distributions (normal, gamma or inverse Gaussian (IG)) are used to simulate cost data. As the two‐stage least squares approach fails to accommodate the correlation between the end points, we examined the effect of different levels of correlation on the methods’ performance; *ρ* takes one of the four values ±0.4 or ±0.8. The final factor is the sample size of the RCT, taking two settings, *n*=100 and *n*=1000. In total, there are 2×3×4×2=48 simulated scenarios.

To generate the data, we begin by simulating *U*∼*N*(0.50,0.25^2^), independently from treatment allocation. *U* represents a pre‐randomization variable that is a common cause of both the outcomes and the probability of non‐compliance, i.e. it is a confounding variable, which we assume is unobserved.

Now, let *S*_*i*_∼Bern(*π*_*s*_) be the random variable denoting whether the *i*th individual switches from allocated active treatment to control. The probability *π*_*s*_ of one‐way non‐compliance with allocated treatment depends on *U* in the following way:

(11) $$\pi_{s}=\left\{\begin{array}{cc} p+0.1, & \text{if}\;u>0.5,\\ p-0.1, & \text{otherwise}, \end{array}\right.$$

where *p* denotes the corresponding average non‐compliance percentage expressed as a probability, i.e. here *p* ∈ {0.3,0.7}. We now generate *D*_*i*_, the random variable of treatment received, as

(12) $$D_{i}=\left\{\begin{array}{cc} Z_{i}, & \text{if either}\;s_{i}=0\;\text{or}\;Z_{i}=0,\\ 1-Z_{i}, & \text{if}\;s_{i}=1\;\text{and}\;Z_{i}=1, \end{array}\right.$$

where *Z*_*i*_ denotes the random allocation for subject *i*.

Then, the means for both outcomes are assumed to depend linearly on treatment received and the unobserved confounder *U* as follows:

(13) $$\mu_{1}=E\left[Y_{1}\right]=1.2+0.4D_{i}+0.16\left(u_{i}-0.5\right),$$

(14) $$\mu_{2}=E\left[Y_{2}\right]=0.5+0.2D_{i}+0.04\left(u_{i}-0.5\right).$$

Finally, the bivariate outcomes are generated by using Gaussian copulas, initially with normal marginals. In subsequent scenarios, we consider gamma or IG marginals for *Y*_1_ and normal marginals for *Y*_2_. The conditional correlation between the outcomes, *ρ*, is set according to the corresponding scenario.

For the scenarios where the end points are assumed to follow a bivariate normal distribution, the variances of the outcomes are set to $\sigma_{1}^{2}=0.2^{2}\;\text{and}\;\sigma_{2}^{2}=0.1^{2}$, whereas, for scenarios with gamma‐ and IG‐distributed *Y*_1_, the shape parameter is *η*=4. For the gamma case, this gives a variance for *Y*_1_ equal to $\sigma_{1}^{2}=0.36$ in the control and $\sigma_{1}^{2}=0.64$ in the intervention group. When *Y*_1_∼IG(*μ*_1_,*η*), the expected variance in the control group is $\sigma_{1}^{2}=0.432$, and $\sigma_{1}^{2}=1.024$ in those receiving the intervention.

The simulated end points represent cost‐effectiveness variables that have been rescaled for computational purposes, with costs divided by 1000, and QALYs by 0.1, such that the true values are £400 (incremental costs) and 0.02 (incremental QALYs) and so, with a threshold value of *λ*=£30 000 per QALY, the true causal INB is £200.

For each simulated scenario, we obtained *M*=2500 sets. For the Bayesian analyses, we use the median of the posterior distribution as the ‘estimate’ of the parameter of interest, and the standard deviation of the posterior distribution as the standard error. Equal‐tailed 95% posterior credible intervals are also obtained. We use the term CI for the Bayesian credible intervals henceforth, to have a unified terminology for both Bayesian and frequentist intervals.

Once the corresponding causal estimate has been obtained in each of the 2500 replicated sets under each scenario in turn, we compute the median bias of the estimates, coverage of 95% CIs, median CI width and root‐mean‐square error (RMSE). We report median bias as opposed to mean bias, because the BFL leads to a posterior distribution of the causal parameters which is Cauchy like (Kleibergen and Zivot, 2003). A method is ‘adequate’, if it results in low levels of bias (median bias 5% or less) with coverage rates within 2.5% of the nominal value.

### 4.1 Implementation

The three‐stage least squares method was fitted by using the systemfit package (Henningsen and Hamann, 2007) in R using FGLS, and the Bayesian methods were run using JAGS (Plummer, 2003) from R (r2jags). Two chains, each with 5000 initial iterations and 1000 burn‐in, were used. The multiple chains allowed for a check of convergence by the degree of their mixing and the initial iterations enabled us to estimate iteration auto‐correlation. A variable number of further 1000‐iteration runs were performed until convergence was reached as estimated by the absolute value of the Geweke statistics for the first 10% and last 50% of iterations in a run being below 2.5. A final additional run of 5000 iterations was performed for each chain to achieve a total sample of 10000 iterations, and a Monte Carlo error of about 1% of the parameter standard error (SE) on which to base the posterior estimates. For model uBGN, an offset of 0.01 was added to the shape parameter *ν*_1_ for the gamma distribution of the cost, to prevent the sampled shape parameter from becoming too close to 0, which may result in infinite densities. See http://wileyonlinelibrary.com/journal/rss-datasets for the JAGS model code for BFL.

### 4.2 Simulation study results

#### 4.2.1 Bias

**Figure 1:**
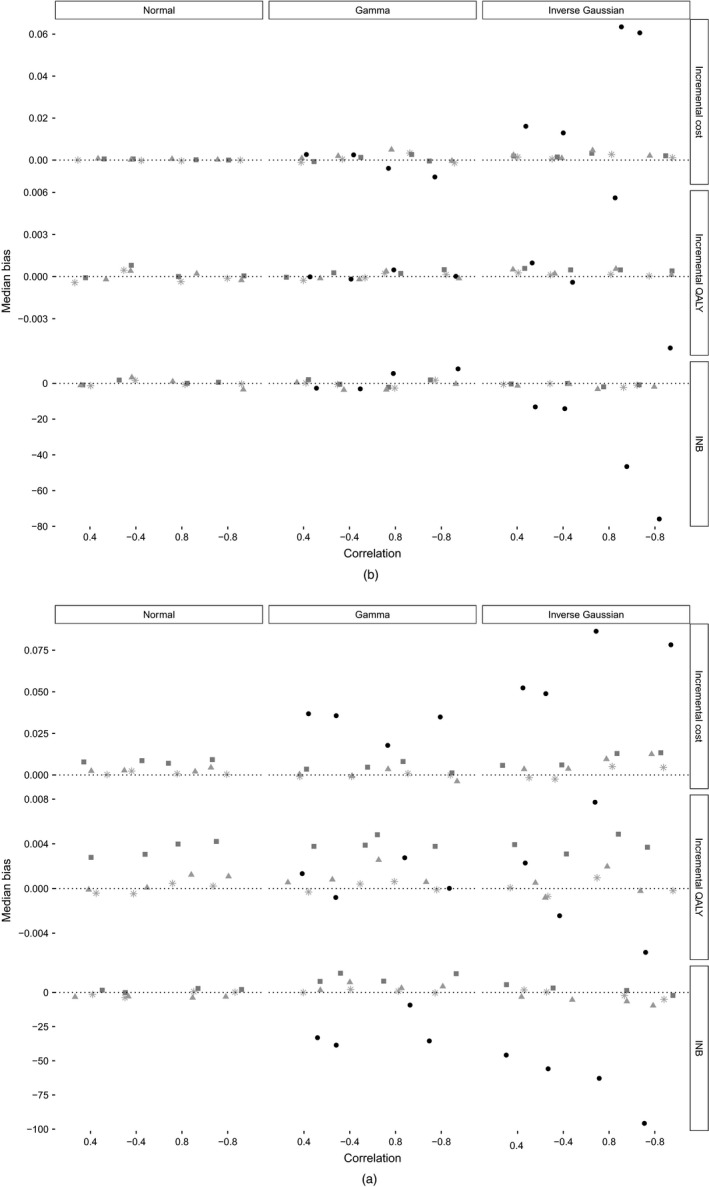
Median bias for scenarios with 30% non‐compliance and sample sizes of (a) *n*=100 and (b) *n*=1000 (results are stratified by cost distribution, and correlation between cost and QALYs; results for two‐stage least squares (not plotted) are identical to those for three‐stage least squares; model uBGN was not applied to normal cost data): 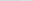, zero bias; 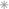, three‐stage least squares; 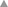, BFL; 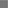, uBN; 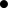, uBGN

Fig. 1 shows the median bias corresponding to scenarios with 30% non‐compliance, by cost distributions (from left to right) and levels of correlation between the two outcomes, for sample sizes of *n*=100 (Fig. 1(a)) and *n*=1000 (Fig. 1(b)). With the larger sample size, for all methods, the bias is negligible with normally distributed costs and remains less than 5% when costs are gamma distributed. However, when costs follow an IG distribution, and the absolute levels of correlation between the end points are high (±0.8), the uBGN approach results in biased estimates: around 10% bias for the estimated incremental cost, and between 20% and 40% for the estimated INB. With the small sample size and when costs follow a gamma or IG distribution, both unadjusted Bayesian methods provide estimates with moderate levels of bias. With 70% non‐compliance (Fig. A3 in the on‐line supplementary file), the unadjusted methods result in important biases which persist even with large sample sizes, especially for scenarios with non‐normal outcomes. For small sample settings, model uBN reports positive bias (10–20%) in the estimation of incremental QALYs, and the resulting INB, irrespectively of the cost distribution. The uBGN method reports relatively unbiased estimates of the QALYs, but large positive bias (up to 60%) in the estimation of costs and, hence, there is substantial bias in the estimated INB (up to 200%). The unadjusted Bayesian methods ignore the positive correlation between the confounding variable and both the treatment received and the outcome. These methods therefore provide estimates of the causal effects that exceed the true values, i.e. have a positive bias. By contrast, the BFL and the three‐stage least squares methods provide estimates with low levels of bias across most settings.

#### 4.2.2 Confidence interval coverage and width

Table 2 presents the results for CI coverage and width, for scenarios with a sample size of *n*=100, absolute levels of correlation between the end points of 0.4 and 30% non‐compliance. All other results are shown in the on‐line supplementary file. The two‐stage least squares method INB ignores the correlation between costs and QALYs, and thus, depending on the direction of this correlation, two‐stage least squares reports CI coverage that is above (positive correlation) or below (negative correlation) nominal levels. This divergence from nominal levels increases with higher absolute levels of correlation (see the supplementary file, Table A6).

**Table 2:** CI coverage rates CR and median width for incremental cost, QALYs and INB, across scenarios with 30% non‐compliance, sample size *n*=100 and moderate correlation *ρ* between outcomes and even rows to negative^a^

	*ρ*	*Results for*		*Results for*		*Results for*		*Results for*		*Results for*	
		*two‐stage*		*three‐stage*		*uBN*		*uBGN*		*BFL*	
		*least squares*		*least squares*							
		*CR*	*CI width*	*CR*	*CI width*	*CR*	*CI width*	*CR*	*CI width*	*CR*	*CI width*
*Y*_1_∼*N*											
Cost	0.4	0.952	0.228	0.952	0.228	0.992	0.312			0.988	0.299
	−0.4	0.952	0.229	0.952	0.229	0.993	0.325			0.986	0.297
QALYs	0.4	0.946	0.112	0.946	0.112	0.988	0.155			0.950	0.121
	−0.4	0.950	0.113	0.950	0.113	0.992	0.163			0.950	0.121
INB	0.4	0.988	405	0.953	319	0.982	398			0.966	376
	−0.4	0.900	409	0.948	475	0.951	509			0.962	525
***Y*_1_∼*G***											
Cost	0.4	0.952	0.756	0.952	0.756	0.955	0.815	0.941	0.818	0.954	0.823
	−0.4	0.942	0.759	0.942	0.759	0.949	0.828	0.936	0.822	0.945	0.811
QALYs	0.4	0.959	0.113	0.959	0.113	0.993	0.160	0.960	0.122	0.960	0.122
	−0.4	0.959	0.113	0.949	0.113	0.995	0.163	0.954	0.122	0.954	0.122
INB	0.4	0.982	829	0.948	696	0.958	764	0.942	748	0.956	760
	−0.4	0.914	833	0.948	943	0.930	921	0.941	1019	0.951	1014
***Y*_1_∼*IG***											
Cost	0.4	0.951	0.880	0.951	0.880	0.958	0.949	0.904	0.866	0.956	0.945
	−0.4	0.950	0.878	0.950	0.878	0.958	0.951	0.905	0.864	0.954	0.932
QALYs	0.4	0.945	0.112	0.945	0.112	0.991	0.161	0.944	0.120	0.999	0.206
	−0.4	0.954	0.112	0.954	0.112	0.993	0.161	0.952	0.120	0.999	0.204
INB	0.4	0.980	944	0.954	818	0.959	889	0.917	814	0.984	1001
	−0.4	0.917	942	0.947	1049	0.934	1034	0.911	1041	0.971	1203

a uBGN was not applied in settings with normal cost data. uBN, unadjusted Bayesian normal–normal model; uBGN, unadjusted Bayesian gamma–normal models.

The uBN approach results in overcoverage across many settings, with wide CIs. For example, for both levels of non‐compliance and either sample size, when the costs are normal, the CI coverage rates for both incremental costs and QALYs exceed 0.98. The interpretation that was offered by Burgess and Thompson (2012) is also relevant here: model uBN assumes that the treatment received and the outcomes variance structures are uncorrelated and so, when the true correlation is positive, the model overstates the variance and leads to wide CIs. By contrast, the uBGN method results in low CI coverage rates for the estimation of incremental costs, when costs follow an IG distribution. This is because the model incorrectly assumes a gamma distribution, thereby underestimating the variance. The extent of the undercoverage appears to increase with higher absolute values of the correlation between the end points, with coverage as low as 0.68 (incremental costs) and 0.72 (INB) in scenarios where the absolute value of correlation between costs and QALYs is 0.8 (see the supplementary file, Table A7).

The BFL approach reports estimates with CI coverage close to the nominal when the sample size is large, but with excess coverage (greater than 0.975), and relatively wide CI, when the sample size is *n*=100 (see Table 2 for 30% non‐compliance, and Table A4 in the supplementary file for the result corresponding to 70% non‐compliance). By contrast, the three‐stage least squares reports CI coverage within 2.5% of nominal levels for each sample size, level of non‐compliance, cost distribution and level of correlation between costs and QALYs.

#### 4.2.3 Root‐mean‐squared error

Table 3 reports RMSE corresponding to 30% non‐compliance, and *n*=100. The least squares approaches result in lower RMSE than the other methods for the summary statistic of interest, INB. This pattern is repeated across other settings; see the on‐line supplementary file, Tables A10–A16. (The RMSE for the two‐stage least squares and three‐stage least squares estimates is the same for each of the outcomes considered, because the two methods obtain the same point estimate, and hence, by definition, they have the same empirical standard error, even though they have different model‐based standard errors for INB. This is in contrast with the differences observed in the performance of measures based on the CI. The coverage rate and CI width corresponding to these two methods are different for INB, because the confidence intervals are constructed by using the model‐based SE. See the on‐line supplementary file for further details.)

**Table 3:** RMSE for incremental cost, QALYs and INB across scenarios with 30% non‐compliance, moderate correlation between outcomes and sample size *n*=100^a^

	*ρ*	*Results for three‐stage*	*Results*	*Results*	*Results*
		*least squares* b	*for uBN*	*for uBGN*	*for BFL*
*Cost distribution normal*					
Cost	0.4	0.058	0.060		0.059
	−0.4	0.060	0.062		0.061
QALYs	0.4	0.029	0.030		0.030
	−0.4	0.029	0.030		0.030
INB	0.4	83	84		87
	−0.4	125	127		125
*Cost distribution gamma*					
Cost	0.4	0.198	0.202	0.212	0.202
	−0.4	0.200	0.204	0.212	0.203
QALYs	0.4	0.030	0.030	0.030	0.029
	−0.4	0.029	0.030	0.030	0.030
INB	0.4	181	184	193	184
	−0.4	246	251	261	252
*Cost distribution IG*					
Cost	0.4	0.230	0.232	0.252	0.232
	−0.4	0.230	0.232	0.250	0.232
QALYs	0.4	0.029	0.030	0.030	0.030
	−0.4	0.029	0.030	0.030	0.030
INB	0.4	211	214	231	214
	−0.4	273	278	296	278

a uBGN was not applied in settings with normal cost data. Numbers for INB have been rounded to the nearest integer. uBN, unadjusted Bayesian normal–normal model; uBGN, unadjusted Bayesian gamma–normal models.

b The RMSE corresponding to two‐stage least squares is identical to that for three‐stage least squares by definition.

## 5 Results for the motivating example

We now compare the methods in practice by applying them to the REFLUX data set. Only 48% of the individuals have completely observed cost‐effectiveness outcomes: there were 185 individuals with missing QALYs, 166 with missing costs and a further 13 with missing EQ5D_0_ at baseline, with about a third of those with missing outcomes having switched from their allocated treatment. These missing data not only bring more uncertainty to our analysis but, more importantly, unless the missing data are handled appropriately can lead to biased causal estimates (Daniel *et al*., 2012). A complete‐case analysis would be unbiased, albeit inefficient, if the missingness is conditionally independent of the outcomes given the covariates in the model (White and Carlin, 2010), even when the covariates have missing data, as here. (This mechanism is a special case of missingness not at random.) Alternatively, a more plausible assumption is to assume that the missing data are missing at random, i.e. the probability of missingness depends only on the observed data, and use multiple imputation (MI) or a full Bayesian analysis to obtain valid inferences.

Therefore, we use an MI prior to carry out two‐stage least squares and three‐stage least squares analyses. We begin by investigating all the possible associations between the covariates that are available in the data set and the missingness, univariately for costs, QALYs and baseline EQ5D_0_. Covariates which are predictive of both, the missing values and the probability of being missing, are to be included in the imputation model as auxiliary variables, as conditioning on more variables helps to make the missingness at random assumption more plausible. None of the available covariates satisfies these criteria and, therefore, we do not include any auxiliary variables in our imputation models. Thus, we impute total cost, total QALYs and baseline EQ5D_0_, 50 times by chained equations, using predictive mean matching, taking the five nearest neighbours as donors (White *et al*., 2011), including treatment received in the imputation model and stratifying by treatment allocation. We perform two‐stage least squares on costs and QALYs independently and calculate (within MI) SE for INB assuming independence between costs and QALYs. For the three‐stage least squares approach, the model is fitted to both outcomes simultaneously, and the post‐estimation facilities are used to extract the variance–covariance estimate and to compute the estimated INB and its corresponding SE. We also use the CACE estimates of incremental cost and QALYs to obtain the ICER. After applying each method to the 50 MI sets, we combine the results by using Rubin's rules (Rubin, 1987). (Applying IV two‐stage least squares and three‐stage least squares with multiply imputed data sets, and combining the results by using Rubin's rules can be done automatically in Stata (StataCorp., 2015) using  mi estimate, cmdok: ivregress 2sls  and  mi estimate, cmdok: reg3. In R, ivregress can be used with the with.mids command within mice, but systemfit cannot at present be combined with this command, so Rubin's rules must be coded manually. Sample code is available from http://wileyonlinelibrary.com/journal/rss-datasets.)

For the Bayesian approaches, the missing values become extra parameters to model. Since baseline EQ5D_0_ has missing observations, a model for its distribution is added, ${\text{EQ5D}}_{0}∼N\left(\mu_{q0},\sigma_{q0}^{2}\right)$, with a vaguely informative prior for *μ*_*q*0_∼Unif(−0.5,1), and an uninformative prior for $|\sigma_{q0}|∼N\left(0,0.01\right)$. We add two extra lines of code to the models to obtain posterior distributions for INB and ICERs. We centre the outcomes near the empirical mean (except for costs, when modelled as gamma) and rescale the costs (dividing by 1000) to improve mixing and convergence. We use two chains, initially running 15000 iterations with 5000 as burn‐in. After checking visually for auto‐correlation, an extra 10000 iterations are needed to ensure that the density plots of the parameters corresponding to the two chains are very similar, denoting convergence to the stationary distribution. Enough iterations for each chain are kept to make the total effective sample (after accounting for auto‐correlation) equal to 10000. (Multivariate normal nodes cannot be partially observed in JAGS; thus, we run BFL models on all available data within WinBUGs (Lunn *et al*., 2000). An observation with zero costs was set to missing when running the Bayesian gamma model, which requires strictly positive costs.)

Table 4 shows the results for incremental costs, QALYs and INB for the motivating example adjusted for baseline EQ5D_0_. Bayesian posterior distributions are summarized by their median value and 95% credible intervals. The CACEs are similar across the methods, except for model uBGN, where the incremental QALYs’ CACE is nearly halved, resulting in a smaller INB with a CI that includes 0. In line with the simulation results, this would suggest that, where model uBGN is misspecified according to the assumed cost distribution, it can provide a biased estimate of the incremental QALYs.

**Table 4:** REFLUX study: cost‐effectiveness according to ITT and alternative methods for estimating the CACE—incremental costs, QALYs and INB of surgery *versus* medicine

*Method*	*Estimate (95% CI)*
*Incremental cost*	
ITT	1103 (593, 1613)
Two‐stage least squares	1899 (1073, 2724)
Three‐stage least squares	1899 (1073, 2724)
uBN	2960 (2026, 3998)
uBGN	2176 (1356, 3031)
BFL	2030 (1170, 2878)
*Incremental QALYs*	
ITT	0.295 (0.002, 0.589)
Two‐stage least squares	0.516 (0.103, 0.929)
Three‐stage least squares	0.516 (0.103, 0.929)
uBN	0.568 (0.181, 0.971 )
uBGN	0.268 (−0.229,0.759)
BFL	0.511 (0.121,0.947)
*INB*	
ITT	7763 (−1059,16585)
Two‐stage least squares	13587 (1101, 26073)
Three‐stage least squares	13587 (1002, 26173)
uBN	14091 (2485, 26086)
uBGN	5869 (−9204,20740)
BFL	13340 (1406,26315)

†The follow‐up period is 5 years, and treatment switches are defined within the first year post randomization. Costs and INB‐numbers have been rounded to the nearest integer. uBN, unadjusted Bayesian normal–normal model; uBGN, unadjusted Bayesian gamma–normal models.

Comparing the CACEs with the ITT, we see that the incremental cost estimates increase between an ITT and a CACE, as actual receipt of surgery carries with it higher costs that the mere offering of surgery does not. Similarly, the incremental QALYs are larger, meaning that, among compliers, those receiving surgery have a greater gain in quality of life, over the follow‐up period. The CACE for costs is relatively close to the per‐protocol incremental costs that were reported in the original study: £2324 (1780,2848). In contrast, the incremental QALYs according to per protocol on complete cases originally reported was 0.3200 (0.0837,0.5562): considerably smaller than our CACE estimates (Grant *et al*., 2013). The ITT ICER that was obtained after MI was £4135, whereas using causal incremental costs and QALYs the corresponding estimates of the CACE for ICER were £4140 (three‐stage least squares), £5189 (uBN), £5960 (uBGN) and £3948 (BFL). The per‐protocol ICER reported by Grant *et al*. (2013) was obtained on complete cases only and was equal to £7932 per QALY.

These results may be sensitive to the modelling of the missing data. As a sensitivity analysis to the missingness at random assumption, we present the complete‐case analysis in Table A1 in the on‐line supplementary file. The conclusions from the complete‐case analysis are similar to those obtained under missingness at random.

We also explore the sensitivity to choices of priors, by rerunning the BFL analyses using different priors, first for the multivariate precision matrix, keeping the priors for the coefficients normal, and then a second analysis, with uniform priors for the regression coefficient, and an inverse Wishart prior with 6 degrees of freedom and an identity scale matrix, for the precision. The results are not materially changed (see the on‐line supplementary file, Table A2).

The results of the within‐trial CEA suggest that, among compliers, laparoscopy is more cost effective than medical management for patients suffering from gastro‐oesophageal reflux disease. The results are robust to the choice of priors, and to the assumptions about the missing data mechanism. The results for model uBGN differ somewhat from those of the other models and, as our simulations show, the concern is that such unadjusted Bayesian models are prone to bias from model misspecification.

## 6 Discussion

This paper extends existing methods for CEA (Willan *et al*., 2003; Nixon and Thompson, 2005), by providing IV approaches for obtaining causal cost‐effectiveness estimates for RCTs with non‐compliance. The methods that were developed here, however, are applicable to other settings with multivariate continuous outcomes more generally, e.g. RCTs in education, with different measures of attainment being combined into an overall score. To help dissemination, code is available from http://wileyonlinelibrary.com/journal/rss-datasets.

We proposed exploiting existing three‐stage least squares methods and also considered IV Bayesian models, which are extensions of previously proposed approaches for univariate continuous outcomes. Burgess and Thompson (2012) found that the BFL was median unbiased and gave CI coverage that was close to nominal levels, albeit with wider CIs than least squares methods. Their ‘unadjusted Bayesian’ method, which is similar to our uBN approach, assumes that the error term for the model of treatment received on treatment allocated is uncorrelated with the error from the outcome models. This results in bias and affects the CI coverage. Our simulation study shows that, in a setting with multivariate outcomes, the bias can be substantial. A potential solution to this could be to use priors for the error terms that reflect the dependence of the error terms explicitly. For example, Rossi *et al*. (2012) proposed a prior for the errors that explicitly depends on the coefficient *β*_1,0_, the effect of treatment allocation on treatment received, in equation (8). Kleibergen and Zivot (2003) proposed priors that also reflect this dependence explicitly and replicate better the properties of the two‐stage least squares. This is known as the ‘Bayesian two‐stage approach’.

The results of our simulations show that applying two‐stage least squares separately to the univariate outcomes leads to inaccurate 95% CIs around INB, even with moderate levels of correlation between costs and outcomes (±0.4). Across all the settings considered, the three‐stage least squares approach resulted in low levels of bias for INB and, unlike two‐stage least squares, provided CI coverage that was close to nominal levels. BFL performed well with large sample sizes but produced standard deviations which were too large when the sample size was small, as can be seen from the overcoverage, with wide CIs.

The REFLUX study illustrated a common concern in CEA, in that the levels of non‐compliance in the RCT were different, in this case higher, from those in routine practice. The CACEs presented provide the policy maker with an estimate of what the relative cost‐effectiveness would be if all the RCT participants had complied with their assigned treatment, which is complementary to the ITT estimate. Since we judged the IV assumptions for identification likely to hold in this case‐study, we conclude that either three‐stage least squares or BFL provide valid inferences for the CACE of INB. The reanalysis of the REFLUX case‐study also provided the opportunity to investigate the sensitivity to the choice of priors in practice. Here we found that our choice of weakly informative priors, which were relatively flat in the region where the values of the parameters were expected to be, together with samples of at least size 100, had minimal influence on the posterior estimates. We repeated the analysis using different vague priors for the parameters of interest and the corresponding results were not materially changed.

The REFLUX study also illustrated a further complication that may arise in practice, namely that covariate or outcome data are missing. Here we illustrated how the methods for estimating the CACE can also accommodate missing data, under the assumption that the data are missing at random, without including any auxiliary variables in the imputation or Bayesian models. However, more generally, where auxiliary variables are available, these should be included in the imputation or Bayesian models. If the auxiliary variables have missing values themselves, this can be accommodated easily via chained equations MI but, for the Bayesian approach, an extra model for the distribution of the auxiliary variable, given the other variables in the substantive model and the outcome, needs to be added.

We considered here relatively simple frequentist IV methods, namely two‐stage least squares and three‐stage least squares. One alternative approach to the estimation of CACEs for multivariate responses is to use linear structural equation modelling, estimated by the maximum likelihood expectation–maximization algorithm (Jo and Muthén, 2001). Further, we considered only those settings where a linear additive treatment effect is of interest, and the assumptions for identification are met. Where interest lies in systems of simultaneous non‐linear equations with endogeneous regressors, generalized method‐of‐moments or generalized structural equation models can be used to estimate CACEs (Davidson and Mackinnon, 2004).

There are several options to study the sensitivity to departures from the identification assumptions. For example, if the exclusion restriction does not hold, a Bayesian parametric model can use priors on the non‐zero direct effect of randomization on the outcome for identification (Conley *et al*., 2012; Hirano *et al*., 2000). Since the models are only weakly identified, the results would depend strongly on the parametric choices for the likelihood and the prior distributions. In the frequentist IV framework, such modelling is also possible; see Baiocchi *et al*. (2014) for an excellent tutorial on how to conduct sensitivity analysis to violations of the exclusion restriction and monotonicity assumptions. Alternatively, violations of the exclusion restriction can also be handled by using baseline covariates to model the probability of compliance directly, within structural equation modelling via the maximum likelihood expectation–maximization framework (Jo, 2002a,b).

Settings where the instrument is only weakly correlated with the endogenous variable have not been considered here, because, for binary non‐compliance with binary allocation, the percentage of one‐way non‐compliance would need to be in excess of 85%, for the *F*‐statistic of the randomization instrument to be less than 10; the traditional cut‐off beneath which an instrument is regarded as ‘weak’. Such levels of non‐compliance are not realistic in practice, with the reported median non‐compliance equal to 12% (Zhang *et al*., 2014). Nevertheless, Bayesian IV methods have been shown to perform better than two‐stage least squares methods when the instrument is weak (Burgess and Thompson, 2012).

Also, for simplicity, we restricted our analysis of the case‐study to missingness at random and complete‐cases assumptions. Sensitivity to departures from these assumptions is beyond the scope of this paper, but researchers should be aware of the need to think carefully about the possible causes of missingness, and to conduct sensitivity analysis under missingness not at random, assuming plausible differences in the distributions of the observed and the missing data. When addressing the missing data through Bayesian methods, the posterior distribution can be sensitive to the choice of prior distribution, especially with a large amount of missing data (Hirano *et al*., 2000).

Future research directions could include exploiting the additional flexibility of the Bayesian framework to incorporate informative priors, perhaps as part of a comprehensive decision modelling approach. The methods that are developed here could also be extended to handle time varying non‐compliance.

## Supporting information

‘Methods for estimating complier‐average causal effects for cost‐effectiveness analysis’.

## References

[rssa12294-bib-0001] Angrist, J. D. , Imbens, G. W. and Rubin, D. B. (1996) Identification of causal effects using instrumental variables. J. Am. Statist. Ass., 91, 444–455.

[rssa12294-bib-0002] Angrist, J. D. and Pischke, J. S. (2008) Mostly Harmless Econometrics: an Empiricist's Companion. Princeton: Princeton University Press.

[rssa12294-bib-0003] Baiocchi, M. , Cheng, J. and Small, D. S. (2014) Instrumental variable methods for causal inference. Statist. Med., 33, 2297–2340. 10.1002/sim.6128 PMC4201653 24599889

[rssa12294-bib-0004] Brilleman, S. , Metcalfe, C. , Peters, T. and Hollingsworth, W. (2015) The reporting of treatment non‐adherence and its associated impact on economic evaluations conducted alongside randomised trials: a systematic review. Val. Hlth, 19, 99–108. 10.1016/j.jval.2015.07.009 26797242

[rssa12294-bib-0005] Burgess, S. and Thompson, S. G. (2012) Improving bias and coverage in instrumental variable analysis with weak instruments for continuous and binary outcomes. Statist. Med., 31, 1582–1600. 10.1002/sim.4498 22374818

[rssa12294-bib-0006] Clarke, P. S. and Windmeijer, F. (2012) Instrumental variable estimators for binary outcomes. J. Am. Statist. Ass., 107, 1638–1652.

[rssa12294-bib-0007] Conley, T. G. , Hansen, C. B. and Rossi, P. E. (2012) Plausibly exogenous. Rev. Econ. Statist., 94, 260–272.

[rssa12294-bib-0008] Daniel, R. M. , Kenward, M. G. , Cousens, S. N. and De Stavola, B. L. (2012) Using causal diagrams to guide analysis in missing data problems. Statist. Meth. Med. Res., 21, 243–256. 10.1177/0962280210394469 21389091

[rssa12294-bib-0009] Davidson, R. and MacKinnon, J. G. (2004) Economic Theory and Methods. New York: Oxford University Press.

[rssa12294-bib-0010] Didelez, V. , Meng, S. and Sheehan, N. (2010) Assumptions of IV methods for observational epidemiology. Statist. Sci., 25, 22–40.

[rssa12294-bib-0011] Dodd, S. , White, I. and Williamson, P. (2012) Nonadherence to treatment protocol in published randomised controlled trials: a review. Trials, 13, article 84. 22709676 10.1186/1745-6215-13-84 PMC3492022

[rssa12294-bib-0012] Gelman, A. and Hill, J. (2006) Data Analysis using Regression and Multilevel/Hierarchical Models. Cambridge: Cambridge University Press.

[rssa12294-bib-0013] Grant, A. M. , Boachie, C. , Cotton, S. C. , Faria, R. , Bojke, L. and Epstein, D. (2013) Clinical and economic evaluation of laparoscopic surgery compared with medical management for gastro‐oesophageal reflux disease: a 5‐year follow‐up of multicentre randomised trial (the REFLUX trial). Hlth Technol. Assessmnt, 17, no. 22. 10.3310/hta17220 PMC4781276 23742987

[rssa12294-bib-0014] Grant, A. , Wileman, S. , Ramsay, C. , Boyke, L. , Epstein, D. and Sculpher, M. (2008) The effectiveness and cost‐effectiveness of minimal access surgery amongst people with gastro‐oesophageal reflux disease—a UK collaborative study: the REFLUX trial. Hlth Technol. Assessmnt, 12, no. 31. 10.3310/hta12310 18796263

[rssa12294-bib-0015] Greene, W. (2002) Econometric Analysis. Englewood Cliffs: Prentice Hall.

[rssa12294-bib-0501] Henningsen, A. and Hamann, J. D. (2007) systemfit: a package for estimating systems of simultaneous equations in R. J. Statist. Softwr., 23, no. 4, 1–40.

[rssa12294-bib-0017] Hirano, K. , Imbens, G. W. , Rubin, D. B. and Zhou, X. H. (2000) Assessing the effecr of an influenza vaccine in an encouragement design. Biostatistics, 1, 69–88. 12933526 10.1093/biostatistics/1.1.69

[rssa12294-bib-0018] Hoch, J. S. , Briggs, A. H. and Willan, A. R. (2002) Something old, something new, something borrowed, something blue: a framework for the marriage of health econometrics and costeffectiveness analysis. Hlth Econ., 11, 415–430. 10.1002/hec.678 12112491

[rssa12294-bib-0019] Hughes, D. , Charles, J. , Dawoud, D. , Edwards, R. T. , Holmes, E. , Jones, C. , Parham, P. , Plumpton, C. , Ridyard, C. , Lloyd‐Williams, H. , Wood, E. and Yeo, S. T. (2016) Conducting economic evaluations alongside randomised trials: current methodological issues and novel approaches. Pharmacoeconomics, 34, article 447. 26753558 10.1007/s40273-015-0371-y

[rssa12294-bib-0020] Imbens, G. W. and Angrist, J. D. (1994) Identification and estimation of local average treatment effects. Econometrica, 62, 467–475.

[rssa12294-bib-0022] Jo, B. (2002a) Estimating intervention effects with noncompliance: alternative model specifications. J. Educ. Behav. Statist., 27, 385–420.

[rssa12294-bib-0023] Jo, B. (2002b) Model misspecification sensitivity analysis in estimating causal effects of interventions with noncompliance. Statist. Med., 21, 3161–3181. 10.1002/sim.1267 12375297

[rssa12294-bib-0024] Jo, B. and Muthén, B. O. (2001) Modeling of intervention effects with noncompliance: a latent variable approach for randomised trials In New Developments and Techniques in Structural Equation Modeling (eds Marcoulides G. A. and Schumacker R. E.), pp. 57–87. Mahwah: Erlbaum.

[rssa12294-bib-0025] Kleibergen, F. and Zivot, E. (2003) Bayesian and classical approaches to instrumental variable regression. J. Econmetr., 114, 29–72.

[rssa12294-bib-0026] Lancaster, T. (2004) Introduction to Modern Bayesian Econometrics. Chichester: Wiley.

[rssa12294-bib-0027] Latimer, N. R. , Abrams, K. , Lambert, P. , Crowther, M. , Wailoo, A. , Morden, J. , Akehurst, R. and Campbell, M. (2014) Adjusting for treatment switching in randomised controlled trials—a simulation study and a simplified two‐stage method. Statist. Meth. Med. Res., 25, 724–751. 10.1177/0962280214557578 25416688

[rssa12294-bib-0502] Lunn, D. J. , Thomas, A. , Best, N. and Spiegelhalter, D. (2000) WinBUGS—a Bayesian modelling framework: concepts, structure and extensibility. Statist. Comput., 10, 325–337.

[rssa12294-bib-0028] Mantopoulos, T. , Mitchell, P. M. , Welton, N. J. McManus, R. and Andronis, L. (2016) Choice of statistical model for cost‐effectiveness analysis and covariate adjustment: empirical application of prominent models and assessment of their results. Eur. J. Hlth Econ., 17, 927–938. 10.1007/s10198-015-0731-8 26445961

[rssa12294-bib-0029] National Institute for Health and Care Excellence (2013) Guide to the Methods of Technology Appraisal. London: National Institute for Health and Care Excellence. 27905712

[rssa12294-bib-0030] Nixon, R. M. and Thompson, S. G. (2005) Methods for incorporating covariate adjustment, sub‐group analysis and between‐centre differences into cost‐effectiveness evaluations. Hlth Econ., 14, 1217–1229. 10.1002/hec.1008 15945043

[rssa12294-bib-0503] Plummer, M. (2003) JAGS: a program for analysis of Bayesian graphical models using Gibbs sampling In Proc. 3rd Int. Wrkshp Distributed Statistical Computing, Vienna, March 20th–22nd (eds Hornik K., Leisch F. and Zeileis A.).

[rssa12294-bib-0031] Rossi, P. , Allenby, G. and McCulloch, R. (2012) Bayesian Statistics and Marketing. Chichester: Wiley.

[rssa12294-bib-0032] Rubin, D. (1987) Multiple Imputation for Nonresponse in Surveys. Chichester: Wiley.

[rssa12294-bib-0033] Schmidt, P. (1990) Three‐stage least squares with different instruments for different equations. J. Econmetr., 43, 389–394.

[rssa12294-bib-0505] StataCorp . (2015) Stata Statistical Software: Release 14. College Station: StataCorp.

[rssa12294-bib-0034] Terza, J. V. , Basu, A. and Rathouz, P. J. (2008) Two‐stage residual inclusion estimation: addressing endogeneity in health econometric modeling. J. Hlth Econ., 27, 531–543. 10.1016/j.jhealeco.2007.09.009 PMC2494557 18192044

[rssa12294-bib-0035] White, I. R. and Carlin, J. B. (2010) Bias and efficiency of multiple imputation compared with complete‐case analysis for missing covariate values. Statist. Med., 28, 2920–2931. 10.1002/sim.3944 20842622

[rssa12294-bib-0036] White, I. R. , Royston, P. and Wood, A. M. (2011) Multiple imputation using chained equations: issues and guidance for practice. Statist. Med., 30, 377–399. 10.1002/sim.4067 21225900

[rssa12294-bib-0037] Willan, A. R. (2006) Statistical analysis of cost‐effectiveness data from randomised clinical trials. Exprt Revisn Pharmecon. Outcmes Res., 6, 337–346. 10.1586/14737167.6.3.337 20528526

[rssa12294-bib-0038] Willan, A. R. , Briggs, A. and Hoch, J. (2004) Regression methods for covariate adjustment and subgroup analysis for non‐censored cost‐effectiveness data. Hlth Econ., 13, 461–475. 10.1002/hec.843 15127426

[rssa12294-bib-0039] Willan, A. R. , Chen, E. , Cook, R. and Lin, D. (2003) Incremental net benefit in randomized clinical trials with quality‐adjusted survival. Statist. Med., 22, 353–362. 10.1002/sim.1347 12529868

[rssa12294-bib-0040] Zellner, A. (1962) An efficient method of estimating seemingly unrelated regressions and tests for aggregation bias. J. Am. Statist. Ass., 57, 348–368.

[rssa12294-bib-0041] Zellner, A. and Huang D. S. (1962) Further properties of efficient estimators for seemingly unrelated regression equations. Int. Econ. Rev., 3, 300–313.

[rssa12294-bib-0042] Zellner, A. and Theil, H. (1962) Three‐stage least squares: simultaneous estimation of simultaneous equations. Econometrica, 30, 54–78.

[rssa12294-bib-0043] Zhang, Z. , Peluso, M. J. , Gross, C. P. , Viscoli, C. M. and Kernan, W. N. (2014) Adherence reporting in randomized controlled trials. Clin. Trials, 11, 195–204. 24357665 10.1177/1740774513512565

