# Supplementary material for: Methods for estimating complier average causal effects for cost‐effectiveness analysis
Source: J R Stat Soc Ser A Stat Soc. 2017 May 24;181(1):277–97. doi: 10.1111/rssa.12294 (PMC5763423; doi:10.1111/rssa.12294)
Supplement: Supplementary file 1 — ‘Methods for estimating complier‐average causal effects for cost‐effectiveness analysis’. [file RSSA-181-277-s001.pdf]

Methods for estimating complier-average causal effects for cost-effectiveness analysis

K. DiazOrdaz, A. J. Franchini, R. Grieve

1 Sensitivity analyses for the REFLUX RCT

Our motivating example trial had over 50% missing cost-effectiveness outcomes, with a few missing data in the baseline covariate. As primary analysis, we performed MI and full Bayesian analyses, that intrinsically obtain a predictive posterior distribution for these missing values, and reported the results of these analyses in the main text.

As a sensitivity to the MAR assumption, we present here the complete case analysis (N=172) of the REFLUX RCT, adjusting for baseline EQ5D. The results are valid if conditional on baseline EQ5D, the missing data mechanism is independent of the outcomes.

Table A1: Estimated cost-effectiveness for the REFLUX RCT, over a time horizon of five years according to ITT and alternative methods for estimating the CACE based on complete cases. Treatment switches are defined within the first year post randomisation. Costs and INB numbers rounded to the nearest integer.

**Incremental costs, QALYs and INB of surgery vs medicine**		
Outcome	Method	estimate (95% CI)
Incremental cost		
	ITT	$1626\ (1098, 2153)$
	2sls	$2102\ (1484, 2720)$
	3sls	$2102\ (1484, 2720)$
	uBN	$2112\ (1401, 2879)$
	uBGN	$2195\ (1481, 3065)$
	BFL	$2081\ (1457, 2705)$
Incremental QALYs		
	ITT	$0.319\ (-0.055, 0.694)$
	2sls	$0.412\ (0.137, 0.688)$
	3sls	$0.412\ (0.137, 0.688)$
	uBN	$0.413\ (0.136, 0.704)$
	uBGN	$0.285\ (-0.066, 0.629)$
	BFL	$0.414\ (0.135, 0.703)$
INB		
	ITT	$7948.\ (-3259, 19155)$
	2sls	$10275\ (1997, 18554)$
	3sls	$10275\ (1828, 18724)$
	uBN	$10280,\ (1947, 18980)$
	uBGN	$6362\ -4497, 16941)$
	BFL	$10353\ (1789, 19165)$

As can be seen in Table A1, the conclusions each model reaches are not substantially changed from those under MAR (either using MI or full Bayesian), presented in the main text.

1.1 Sensitivity analysis of the BFL to choice of priors

We now study the sensitivity of the BFL model to the choice of priors for the parameters in the model. Recall that this model can be written as

$$\begin{pmatrix} D_i \\ Y_{1i} \\ Y_{2i} \end{pmatrix} \sim N\left\{\begin{pmatrix} \mu_{0i} \\ \mu_{1i} \\ \mu_{2i} \end{pmatrix}, \Sigma\right\} \qquad \Sigma = \begin{pmatrix} \sigma_0^2 & s_{01} & s_{02} \\ s_{01} & \sigma_1^2 & s_{12} \\ s_{02} & s_{12} & \sigma_2^2 \end{pmatrix} \tag{1}$$

where $s_{ij} = \text{cov}(Y_i, Y_j)$, and

$$\begin{aligned} \mu_{0i} &= \beta_{0,0} + \beta_{1,0} Z_i \\ \mu_{1i} &= \beta_{0,1} + \beta_{1,1}\beta_{1,0} Z_i \\ \mu_{2i} &= \beta_{0,2} + \beta_{1,2}\beta_{1,0} Z_i \end{aligned} \tag{2}$$

Originally, we chose normal priors for the regression coefficients, $\beta_{m,j} \sim N(0, 10^2)$, for $j \in \{0,1,2\}, m \in \{0,1\}$, and a Wishart prior for the inverse of Σ (Gelman and Hill, 2006).

Here, we first vary the prior distribution for Σ, and assume a structured covariance matrix (see Congdon (2007) example 5.8, and Lunn et al. (2012), example 9.1.4). Thus, we write:

$$\Sigma = \begin{pmatrix} s_{00}s_{00} & \rho_1 s_{00}s_{11} & \rho_2 s_{00}s_{22} \\ \rho_1 s_{00}s_{11} & s_{11}s_{11} & \rho_3 s_{11}s_{22} \\ \rho_2 s_{00}s_{22} & \rho_3 s_{11}s_{22} & s_{22}s_{22} \end{pmatrix} \tag{3}$$

and assumed the following priors

$$s_{ij} \sim N(0, 10^2), \qquad \rho_j \sim \text{Unif}[-1, 1] \tag{4}$$

In a secondary sensitivity analysis, we assume a Wishart prior for Σ as before, but used uniform priors for the regression coefficients, $\beta_{m,j} \sim \text{Unif}[-10, 10]$, for $j \in \{0,1,2\}, m \in \{0,1\}$. We did these changes on both available cases and complete cases. The results corresponding to INB are reported in Table A2.

Table A2: Results from sensitivity analysis of the BFL to prior specifications for the INB of the motivating example: CEA within the REFLUX trial. INB reported to the nearest integer, in Great British Pounds (GBP).

Data	Priors	INB estimate	95% CI
Available cases	Structured precision matrix[1] normal priors for $\beta_{m,j}$	10421	$(1878, 19187)$
Available cases	Wishart prior for the precision Unif prior for $\beta_{m,j}$	10405	$(1733, 19330)$
Complete cases	Structured precision matrix normal priors for $\beta_{m,j}$	10425	$(1792, 19156)$
Complete cases	Wishart prior for the precision Unif prior for $\beta_{m,j}$	10428	$(1894, 19279)$

[1] Following the specifications of equation (4) in this Appendix.

2 Supplementary Results from the simulations

Figure A1: Median Bias for scenarios with 70% non-compliance and sample sizes of (a) $n = 100$ (top) and (b) $n = 1000$. Results are stratified by cost distribution, and correlation between cost and QALYs. The dotted line represents zero bias. Results for 2sls (not plotted) are identical to those for 3sls; uBGN was not applied to Normal cost data.

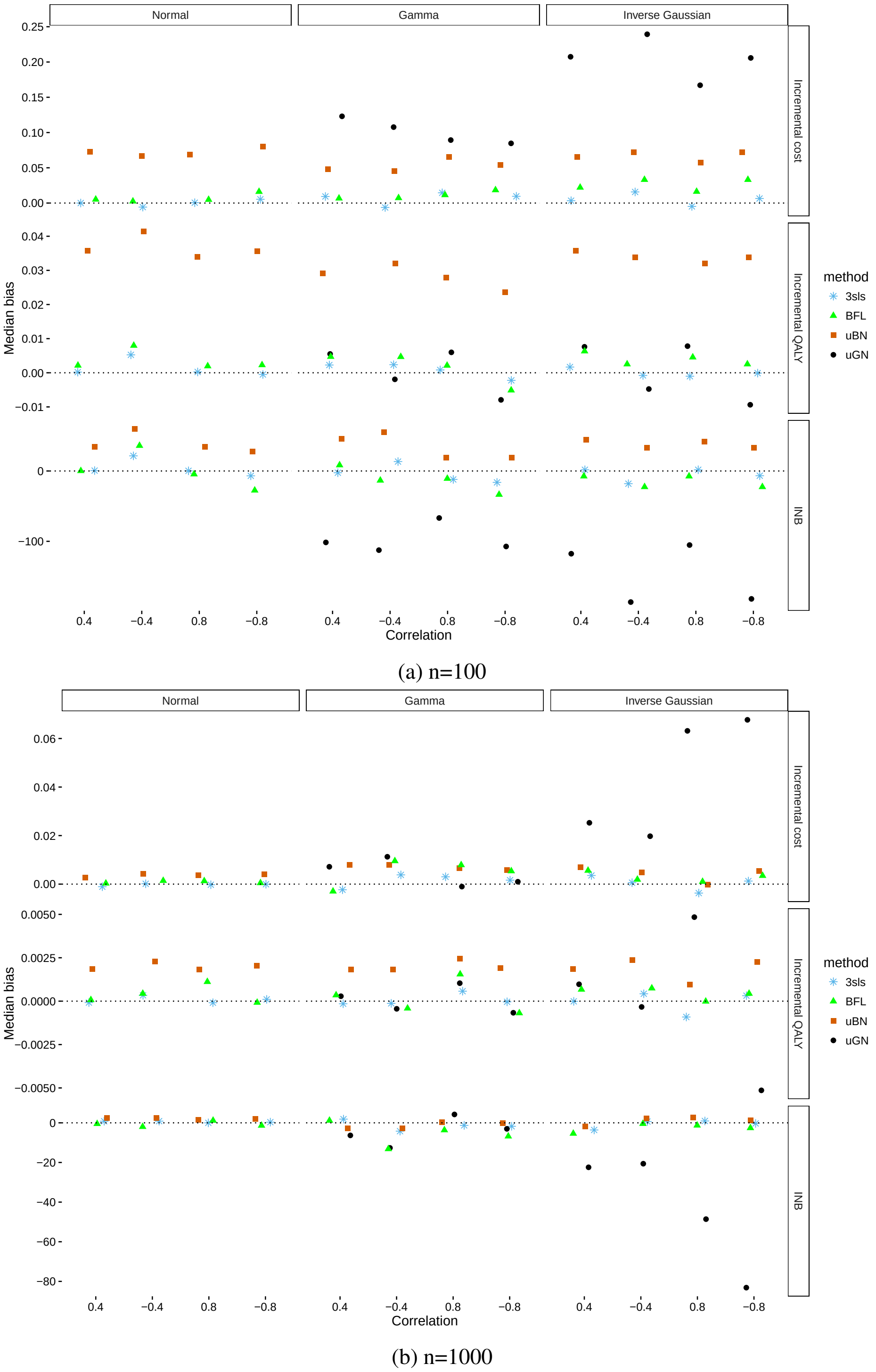

(a) n=100

(b) n=1000

Table A3: CI Coverage rates and median width for incremental Cost, QALYs, and INB, across scenarios with 30% non-compliance, moderate correlation between outcomes and sample size $n = 1000$. uBGN was not applied in settings with normal cost data.

			2sls		3sls		uBN		uBGN		BFL	
**Cost distribution**		ρ	**coverage**	**width**	**coverage**	**width**	**coverage**	**width**	**coverage**	**width**	**coverage**	**width**
**Normal**	**Cost**											
		0.4	0.949	0.072	0.949	0.072	0.989	0.094			0.956	0.075
		-0.4	0.957	0.072	0.957	0.072	0.992	0.097			0.963	0.075
	**QALYs**											
		0.4	0.942	0.036	0.942	0.036	0.982	0.046			0.937	0.035
		-0.4	0.947	0.036	0.947	0.036	0.991	0.048			0.944	0.035
	**INB**											
		0.4	0.986	129	0.951	102	0.977	120			0.950	101
		-0.4	0.920	129	0.956	150	0.962	155			0.949	149
**Gamma**	**Cost**											
		0.4	0.949	0.240	0.949	0.240	0.955	0.246	0.943	0.235	0.951	0.242
		-0.4	0.953	0.240	0.953	0.240	0.958	0.248	0.945	0.235	0.954	0.243
	**QALYs**											
		0.4	0.947	0.036	0.947	0.036	0.990	0.048	0.946	0.036	0.943	0.035
		-0.4	0.950	0.036	0.950	0.036	0.993	0.048	0.949	0.036	0.950	0.036
	**INB**											
		0.4	0.985	262	0.950	221	0.957	229	0.944	218	0.949	221
		-0.4	0.920	262	0.952	297	0.932	276	0.946	292	0.954	301
**Inverse Gaussian**	**Cost**											
		0.4	0.949	0.283	0.949	0.283	0.958	0.290	0.902	0.248	0.947	0.281
		-0.4	0.952	0.283	0.952	0.283	0.957	0.291	0.901	0.249	0.946	0.277
	**QALYs**											
		0.4	0.954	0.036	0.954	0.036	0.990	0.048	0.942	0.035	0.967	0.038
		-0.4	0.949	0.036	0.949	0.036	0.989	0.048	0.942	0.035	0.961	0.038
	**INB**											
		0.4	0.976	303	0.956	263	0.958	268	0.914	235	0.958	268
		-0.4	0.922	302	0.950	336	0.932	315	0.906	300	0.947	333

Table A4: CI Coverage rates and median width for incremental Cost, QALYs, and INB, across scenarios with 70% non-compliance, moderate correlation between outcomes and sample size $n = 100$. uBGN was not applied in settings with normal cost data.

			2sls		3sls		uBN		uBGN		BFL	
**Cost distribution**		ρ	**coverage**	**width**	**coverage**	**width**	**coverage**	**width**	**coverage**	**width**	**coverage**	**width**
**Normal**	**Cost**											
		0.4	0.968	0.550	0.968	0.550	0.988	1.33			0.991	1.13
		-0.4	0.966	0.545	0.966	0.545	0.990	1.30			0.985	1.08
	**QALYs**											
		0.4	0.968	0.271	0.968	0.271	0.988	0.665			0.964	0.445
		-0.4	0.964	0.269	0.964	0.269	0.986	0.665			0.954	0.435
	**INB**											
		0.4	0.992	981	0.966	771	0.985	1453			0.981	1411
		-0.4	0.917	973	0.966	1131	0.957	1788			0.958	1912
**Gamma**	**Cost**											
		0.4	0.966	1.68	0.966	1.68	0.964	2.640	0.944	2.21	0.960	2.73
		-0.4	0.969	1.69	0.969	1.69	0.965	2.690	0.950	2.21	0.964	2.71
	**QALYs**											
		0.4	0.972	0.267	0.972	0.267	0.994	0.624	0.959	0.348	0.965	0.42
		-0.4	0.965	0.269	0.965	0.269	0.987	0.625	0.954	0.351	0.954	0.42
	**INB**											
		0.4	0.988	1870	0.966	1557	0.964	2456	0.947	2041	0.970	2503
		-0.4	0.939	1876	0.971	2138	0.947	2984	0.958	2774	0.962	3344
**Inverse Gaussian**	**Cost**											
		0.4	0.965	1.92	0.965	1.92	0.967	3.04	0.912	2.29	0.972	3.31
		-0.4	0.958	1.90	0.958	1.90	0.966	3.05	0.910	2.30	0.968	3.37
	**QALYs**											
		0.4	0.967	0.268	0.967	0.268	0.988	0.637	0.948	0.355	1	0.766
		-0.4	0.966	0.271	0.966	0.271	0.990	0.631	0.945	0.359	0.999	0.774
	**INB**											
		0.4	0.990	2082	0.961	1777	0.963	2827	0.912	2157	0.991	3554
		-0.4	0.936	2071	0.970	2335	0.912	3389	0.924	2855	0.979	4819

Table A5: CI Coverage rates and median width for incremental Cost, QALYs, and INB, across scenarios with 70% non-compliance, moderate correlation between outcomes and sample size $n = 1000$. uBGN was not applied in settings with normal cost data.

			2sls		3sls		uBN		uBGN		BFL	
**Cost distribution**		ρ	**coverage**	**width**	**coverage**	**width**	**coverage**	**width**	**coverage**	**width**	**coverage**	**width**
**Normal**	**Cost**											
		0.4	0.954	0.169	0.954	0.169	0.986	0.223			0.958	0.178
		-0.4	0.953	0.169	0.953	0.169	0.991	0.230			0.960	0.179
	**QALYs**											
		0.4	0.946	0.083	0.946	0.083	0.986	0.111			0.942	0.084
		-0.4	0.953	0.083	0.953	0.083	0.992	0.115			0.947	0.083
	**INB**											
		0.4	0.986	301	0.948	238	0.976	287			0.944	240
		-0.4	0.909	301	0.944	349	0.953	368			0.942	356
**Gamma**	**Cost**											
		0.4	0.952	0.526	0.952	0.526	0.958	0.556	0.939	0.523	0.952	0.541
		-0.4	0.952	0.525	0.952	0.525	0.958	0.556	0.943	0.524	0.952	0.541
	**QALYs**											
		0.4	0.951	0.083	0.951	0.0832	0.995	0.115	0.947	0.085	0.949	0.084
		-0.4	0.955	0.083	0.955	0.0832	0.995	0.115	0.954	0.084	0.955	0.085
	**INB**											
		0.4	0.980	582	0.954	486	0.932	626	0.946	487	0.951	496
		-0.4	0.916	581	0.953	661	0.932	626	0.944	662	0.953	678
**Inverse Gaussian**	**Cost**											
		0.4	0.952	0.595	0.952	0.595	0.959	0.621	0.909	0.538	0.950	0.603
		-0.4	0.946	0.955	0.599	0.955	0.599	0.629	0.910	0.542	0.948	0.599
	**QALYs**											
		0.4	0.943	0.083	0.943	0.083	0.991	0.114	0.938	0.084	0.959	0.0918
		-0.4	0.083	0.946	0.083	0.96	0.988	0.115	0.942	0.084	0.957	0.0905
	**INB**											
		0.4	0.975	645	0.952	552	0.954	577	0.918	510	0.953	571
		-0.4	0.920	649	0.952	728	0.93	692	0.914	668	0.950	736

Table A6: CI Coverage rates and median width for incremental Cost, QALYs, and INB, across scenarios with 30% non-compliance, high correlation between outcomes and sample size $n = 100$. uBGN was not applied in settings with normal cost data.

			2sls		3sls		uBN		uBGN		BFL	
**Cost distribution**		ρ	**coverage**	**width**	**coverage**	**width**	**coverage**	**width**	**coverage**	**width**	**coverage**	**width**
**Normal**	**Cost**											
		0.8	0.951	0.229	0.951	0.229	0.984	0.299			0.987	0.299
		-0.8	0.946	0.228	0.946	0.228	0.992	0.324			0.986	0.295
	**QALYs**											
		0.8	0.954	0.113	0.954	0.113	0.985	0.148			0.958	0.122
		-0.8	0.949	0.112	0.949	0.112	0.992	0.162			0.952	0.121
	**INB**											
		0.8	0.999	408	0.953	207	0.998	373			0.990	279
		-0.8	0.867	407	0.947	533	0.93	514			0.957	585
**Gamma**	**Cost**											
		0.8	0.955	0.757	0.955	0.757	0.947	0.764	0.922	0.681	0.961	0.822
		-0.8	0.946	0.757	0.946	0.757	0.954	0.825	0.947	0.820	0.951	0.817
	**QALYs**											
		0.8	0.952	0.113	0.952	0.113	0.986	0.152	0.928	0.109	0.957	0.123
		-0.8	0.947	0.113	0.947	0.113	0.99	0.162	0.950	0.121	0.954	0.123
	**INB**											
		0.8	0.999	828	0.955	539	0.99	707	0.942	528	0.966	596
		-0.8	0.884	831	0.945	1041	0.901	920	0.882	902	0.95	1118
**Inverse Gaussian**	**Cost**											
		0.8	0.954	0.883	0.954	0.883	0.948	0.894	0.836	0.745	0.961	0.956
		-0.8	0.953	0.884	0.953	0.884	0.961	0.956	0.826	0.750	0.955	0.938
	**QALYs**											
		0.8	0.954	0.113	0.954	0.113	0.989	0.154	0.900	0.108	1	0.204
		-0.8	0.951	0.113	0.951	0.113	0.993	0.163	0.916	0.108	1	0.204
	**INB**											
		0.8	0.994	947	0.952	674	0.978	825	0.837	591	0.990	877
		-0.8	0.893	948	0.953	1155	0.910	1042	0.842	993	0.969	1307

Table A7: CI Coverage rates and median width for incremental Cost, QALYs, and INB, across scenarios with 30% non-compliance, high correlation between outcomes and sample size $n = 1000$.uBGN was not applied in settings with normal cost data.

			2sls		3sls		uBN		uBGN		BFL	
**Cost distribution**		ρ	**coverage**	**width**	**coverage**	**width**	**coverage**	**width**	**coverage**	**width**	**coverage**	**width**
**Normal**	**Cost**											
		0.8	.949	0.072	0.949	0.072	0.985	0.089			0.951	0.074
		-0.8	0.954	0.072	0.954	0.072	0.996	0.097			0.955	0.074
	**QALYs**											
		0.8	0.943	0.036	0.943	0.036 0	0.988	0.044			0.938	0.035
		-0.8	0.952	0.036	0.952	0.036	0.992	0.048			0.951	0.036
	**INB**											
		0.8	1	129	0.95	65.4	1	112			0.946	66.3
		-0.8	0.864	129	0.952	168.4	0.928	154			0.946	170.2
**Gamma**	**Cost**											
		0.8	0.946	0.240	0.946	0.240	0.938	0.230	0.897	0.199	0.944	0.239
		-0.8	0.949	0.240	0.949	0.240	0.955	0.249	0.896	0.202	0.950	0.241
	**QALYs**											
		0.8	0.948	0.036	0.948	0.036	0.983	0.045	0.921	0.032	0.946	0.035
		-0.8	0.952	0.036	0.952	0.036	0.990	0.048	0.925	0.032	0.950	0.036
	**INB**											
		0.8	0.996	263	0.950	172	0.982	213	0.928	153	0.948	172
		-0.8	0.883	263	0.946	329	0.899	277	0.898	275	0.946	330
**Inverse Gaussian**	**Cost**											
		0.8	0.946	0.283	0.946	0.283	0.936	0.271	0.684	0.217	0.942	0.279
		-0.8	0.952	0.283	0.952	0.283	0.955	0.290	0.701	0.221	0.950	0.280
	**QALYs**											
		0.8	0.950	0.0356	0.95	0.0356	0.986	0.0457	0.833	0.032	0.964	0.038
		-0.8	0.944	0.0356	0.944	0.0356	0.990	0.048	0.849	0.032	0.965	0.038
	**INB**											
		0.8	0.994	303	0.952	218	0.972	250	0.722	173	0.954	221
		-0.8	0.89	302	0.947	367	0.904	315	0.722	292	0.946	364

Table A8: CI Coverage rates and median width for incremental Cost, QALYs, and INB, across scenarios with 70% non-compliance, high correlation between outcomes and sample size $n = 100$. uBGN was not applied in settings with normal cost data.

			2sls		3sls		uBN		uBGN		BFL	
**Cost distribution**		ρ	**coverage**	**width**	**coverage**	**width**	**coverage**	**width**	**coverage**	**width**	**coverage**	**width**
**Normal**	**Cost**											
		0.8	0.972	0.546	0.972	0.546	0.982	1.29			0.988	1.09
		-0.8	0.970	0.550	0.970	0.550	0.99	1.36			0.988	1.07
	**QALYs**											
		0.8	0.97	0.269	0.97	0.269	0.982	0.64			0.966	0.443
		-0.8	0.966	0.272	0.966	0.272	0.989	0.662			0.960	0.446
	**INB**											
		0.8	1	975	0.973	495	1	1322			0.993	1032
		-0.8	0.875	981	0.966	1284	0.936	1858			0.957	2096
**Gamma**	**Cost**											
		0.8	0.967	1.68	0.967	1.68	0.95	2.51	0.921	1.82	0.967	2.77
		-0.8	0.965	1.70	0.965	1.70	0.965	2.75	0.904	1.88	0.964	2.80
	**QALYs**											
		0.8	0.969	0.268	0.969	0.268	0.984	0.609	0.935	0.315	0.967	0.430
		-0.8	0.966	0.272	0.966	0.272	0.989	0.626	0.930	0.324	0.958	0.440
	**INB**											
		0.8	1	1871	0.963	1175	0.992	2233	0.947	1397	0.975	1964
		-0.8	0.895	1885	0.965	2383	0.908	3096	0.903	2631	0.954	3867
**Inverse Gaussian**	**Cost**											
		0.8	0.96	1.92	0.96	1.92	0.947	2.92	0.839	2.03	0.966	3.42
		-0.8	0.964	1.91	0.964	1.91	0.966	3.05	0.855	2.03	0.968	3.37
	**QALYs**											
		0.8	0.963	0.271	0.963	0.271	0.983	0.645	0.906	0.322	0.999	0.789
		-0.8	0.973	0.27	0.973	0.270	0.99	0.631	0.918	0.325	0.999	0.774
	**INB**											
		0.8	0.998	2091	0.958	1417	0.981	2654	0.863	1586	0.990	3145
		-0.8	0.902	2075	0.966	2568	0.912	3389	0.866	2755	0.979	4819

Table A9: CI Coverage rates and median width for incremental Cost, QALYs, and INB, across scenarios with 70% non-compliance, high correlation between outcomes and sample size $n = 1000$. uBGN was not applied in settings with normal cost data.

			2sls		3sls		uBN		uBGN		BFL	
**Cost distribution**		ρ	**coverage**	**width**	**coverage**	**width**	**coverage**	**width**	**coverage**	**width**	**coverage**	**width**
**Normal**	**Cost**											
		0.8	0.959	0.169	0.959	0.169	0.983	0.211			0.959	0.175
		-0.8	0.952	0.169	0.952	0.169	0.991	0.230			0.954	0.177
	**QALYs**											
		0.8	0.957	0.083	0.957	0.083	0.983	0.106			0.951	0.084
		-0.8	0.954	0.083	0.954	0.083	0.991	0.115			0.951	0.085
	**INB**											
		0.8	1	302	0.952	153	0.999	269			0.952	158
		-0.8	0.873	302	0.954	395	0.931	369			0.953	406
**Gamma**	**Cost**											
		0.8	0.952	0.527	0.952	0.527	0.938	0.514	0.890	0.442	0.948	0.535
		-0.8	0.950	0.525	0.95	0.525	0.954	0.556	0.904	0.449	0.949	0.542
	**QALYs**											
		0.8	0.958	0.0833	0.958	0.0833	0.986	0.108	0.923	0.076	0.952	0.0841
		-0.8	0.951	0.0832	0.951	0.0832	0.992	0.115	0.930	0.077	0.947	0.0843
	**INB**											
		0.8	0.998	583	0.953	368	0.986	479	0.923	336	0.950	374
		-0.8	0.888	582	0.953	734	0.902	627	0.904	627	0.951	753
**Inverse Gaussian**	**Cost**											
		0.8	0.952	0.599	0.952	0.599	0.938	0.583	0.785	0.474	0.946	0.602
		-0.8	0.959	0.600	0.959	0.600	0.962	0.630	0.786	0.482	0.955	0.604
	**QALYs**											
		0.8	0.948	0.083	0.948	0.083	0.992	0.109	0.891	0.076	0.96	0.091
		-0.8	0.956	0.083	0.956	0.083	0.992	0.115	0.896	0.077	0.968	0.091
	**INB**											
		0.8	0.996	650	0.946	447	0.978	542	0.800	370	0.955	468
		-0.8	0.899	650	0.96	801	0.915	694	0.806	654	0.958	807

Table A10: RMSE for incremental Cost, QALYs and INB across scenarios with 30% non-compliance, high correlation between outcomes and sample size $n = 100$. uBGN was not applied in settings with normal cost data. Numbers for INB have been rounded to the nearest integer..[a]

Cost distribution		ρ	3sls[b]	uBN	uBGN	BFL
**Normal**	**Cost**					
		0.8	0.059	0.060		0.060
		-0.8	0.059	0.061		0.060
	**QALYs**					
		0.8	0.029	0.030		0.030
		-0.8	0.029	0.030		0.030
	**INB**					
		0.8	54	55		56
		-0.8	137	140		139
**Gamma**	**Cost**					
		0.8	0.192	0.196	0.194	0.196
		-0.8	0.196	0.200	0.205	0.200
	**QALYs**					
		0.8	0.029	0.030	0.030	0.030
		-0.8	0.029	0.030	0.030	0.030
	**INB**					
		0.8	138	141	137	141
		-0.8	271	276	281	276
**Inverse Gaussian**	**Cost**					
		0.8	0.226	0.229	0.272	0.229
		-0.8	0.225	0.228	0.272	0.228
	**QALYs**					
		0.8	0.029	0.030	0.032	0.030
		-0.8	0.028	0.029	0.031	0.029
	**INB**					
		0.8	175	178	210	178
		-0.8	291	296	345	296

[a] uBN: unadjusted Bayesian Normal-Normal model;uBGN: unadjusted Bayesian Gamma-Normal models; BFL: Bayesian Full Likelihood

[b] The RMSE corresponding to 2sls is identical to that for 3sls, by definition.

Table A11: RMSE for incremental Cost, QALYs and INB across scenarios with 30% non-compliance, moderate correlation between outcomes and sample size $n = 1000$. uBGN was not applied in settings with normal cost data. Numbers for INB have been rounded to the nearest integer.[a]

Cost distribution		ρ	3sls[b]	uBN	uBGN	BFL
**Normal**	**Cost**					
		0.4	0.018	0.018		0.018
		-0.4	0.018	0.018		0.018
	**QALYs**					
		0.4	0.009	0.009		0.009
		-0.4	0.009	0.009		0.009
	**INB**					
		0.4	26	26		26
		-0.4	38	38		39
**Gamma**	**Cost**					
		0.4	0.061	0.061	0.061	0.061
		-0.4	0.061	0.061	0.061	0.061
	**QALYs**					
		0.4	0.009	0.009	0.009	0.009
		-0.4	0.009	0.009	0.009	0.009
	**INB**					
		0.4	55	55	56	55
		-0.4	75	75	76	75
**Inverse Gaussian**	**Cost**					
		0.4	0.072	0.072	0.075	0.072
		-0.4	0.072	0.072	0.076	0.073
	**QALYs**					
		0.4	0.009	0.009	0.035	0.009
		-0.4	0.009	0.009	0.035	0.009
	**INB**					
		0.4	66	66	69	66
		-0.4	85	86	89	86

[a] uBN: unadjusted Bayesian Normal-Normal model;uBGN: unadjusted Bayesian Gamma-Normal models; BFL: Bayesian Full Likelihood

[b] The RMSE corresponding to 2sls is identical to that for 3sls, by definition.

Table A12: RMSE for incremental Cost, QALYs and INB across scenarios with 30% non-compliance, high correlation between outcomes and sample size $n = 1000$. uBGN was not applied in settings with normal cost data. Numbers for INB have been rounded to the nearest integer.[a]

Cost distribution		ρ	3sls[b]	uBN	uBGN	BFL
**Normal**	**Cost**					
		0.8	0.019	0.019		0.019
		-0.8	0.018	0.018		0.018
	**QALYs**					
		0.8	0.009	0.009		0.009
		-0.8	0.009	0.009		0.009
	**INB**					
		0.8	17	17		17
		-0.8	43	43		44
**Gamma**	**Cost**					
		0.8	0.062	0.062	0.061	0.062
		-0.8	0.062	0.062	0.062	0.062
	**QALYs**					
		0.8	0.009	0.009	0.009	0.009
		-0.8	0.009	0.009	0.009	0.009
	**INB**					
		0.8	44	44	43	44
		-0.8	85	85	84	85
**Inverse Gaussian**	**Cost**					
		0.8	0.073	0.073	0.105	0.073
		-0.8	0.072	0.072	0.103	0.072
	**QALYs**					
		0.8	0.009	0.009	0.011	0.009
		-0.8	0.009	0.009	0.011	0.009
	**INB**					
		0.8	56	56	80	56
		-0.8	94	94	132	94

Table A13: RMSE for incremental Cost, QALYs and INB across scenarios with 70% non-compliance, moderate correlation between outcomes and sample size $n = 100$. uBGN was not applied in settings with normal cost data. Numbers for INB have been rounded to the nearest integer.[a]

Cost distribution		ρ	3sls[b]	uBN	uBGN	BFL
**Normal**	**Cost**					
		0.4	0.145	0.207		0.181
		-0.4	0.146	0.203		0.182
	**QALYs**					
		0.4	0.074	0.104		0.089
		-0.4	0.074	0.109		0.088
	**INB**					
		0.4	212	272		264
		-0.4	309	396		383
**Gamma**	**Cost**					
		0.4	0.467	0.565	0.682	0.559
		-0.4	0.456	0.553	0.636	0.549
	**QALYs**					
		0.4	0.072	0.097	0.082	0.085
		-0.4	0.073	0.099	0.083	0.086
	**INB**					
		0.4	426	520	429	500
		-0.4	570	697	758	687
**Inverse Gaussian**	**Cost**					
		0.4	0.521	0.622	0.946	0.622
		-0.4	0.516	0.635	1.096	0.635
	**QALYs**					
		0.4	0.073	0.094	0.092	0.094
		-0.4	0.074	0.092	0.110	0.092
	**INB**					
		0.4	484	583	632	583
		-0.4	630	865	967	865

Table A14: RMSE for incremental Cost, QALYs and INB across scenarios with 70% non-compliance, high correlation between outcomes and sample size $n = 100$. uBGN was not applied in settings with normal cost data. Numbers for INB have been rounded to the nearest integer.[a]

Cost distribution		ρ	3sls[b]	uBN	uBGN	BFL
**Normal**	**Cost**					
		0.8	0.147	0.201		0.180
		-0.8	0.147	0.209		0.182
	**QALYs**					
		0.8	0.072	0.100		0.086
		-0.8	0.075	0.109		0.090
	**INB**					
		0.8	131	169		169
		-0.8	351	447		425
**Gamma**	**Cost**					
		0.8	0.450	0.543	0.604	0.531
		-0.8	0.454	0.546	0.563	0.538
	**QALYs**					
		0.8	0.072	0.097	0.081	0.086
		-0.8	0.073	0.096	0.084	0.086
	**INB**					
		0.8	313	377	429	368
		-0.8	638	768	758	773
**Inverse Gaussian**	**Cost**					
		0.8	0.532	0.637	0.902	0.637
		-0.8	0.526	0.635	1.010	0.635
	**QALYs**					
		0.8	0.074	0.092	0.100	0.092
		-0.8	0.074	0.092	0.096	0.092
	**INB**					
		0.8	395	468	615	468
		-0.8	704	865	1022	865

Table A15: RMSE for incremental Cost, QALYs and INB across scenarios with 70% non-compliance, moderate correlation between outcomes and sample size $n = 1000$. uBGN was not applied in settings with normal cost data. Numbers for INB have been rounded to the nearest integer.[a]

Cost distribution		ρ	3sls[b]	uBN	uBGN	BFL
**Normal**	**Cost**					
		0.4	0.043	0.043		0.043
		-0.4	0.043	0.043		0.043
	**QALYs**					
		0.4	0.022	0.022		0.022
		-0.4	0.022	0.022		0.022
	**INB**					
		0.4	61	62		62
		-0.4	90	91		91
**Gamma**	**Cost**					
		0.4	0.135	0.135	0.138	0.136
		-0.4	0.134	0.135	0.138	0.135
	**QALYs**					
		0.4	0.021	0.021	0.021	0.021
		-0.4	0.021	0.021	0.021	0.021
	**INB**					
		0.4	124	169	126	125
		-0.4	168	169	172	170
**Inverse Gaussian**	**Cost**					
		0.4	0.151	0.153	0.158	0.153
		-0.4	0.152	0.153	0.158	0.153
	**QALYs**					
		0.4	0.021	0.022	0.022	0.022
		-0.4	0.021	0.022	0.022	0.022
	**INB**					
		0.4	141	142	147	142
		-0.4	185	187	193	187

[a] uBN: unadjusted Bayesian Normal-Normal model;uBGN: unadjusted Bayesian Gamma-Normal models; BFL: Bayesian Full Likelihood

[b] The RMSE corresponding to 2sls is identical to that for 3sls, by definition.

Table A16: RMSE for incremental Cost, QALYs and INB across scenarios with 70% non-compliance, high correlation between outcomes and sample size $n = 1000$. uBGN was not applied in settings with normal cost data. Numbers for INB have been rounded to the nearest integer.[a]

Cost distribution		ρ	3sls[b]	uBN	uBGN	BFL
**Normal**	**Cost**					
		0.8		0.043		0.043
		-0.8	0.043	0.043		0.043
	**QALYs**					
		0.8	0.021	0.021		0.021
		-0.8	0.021	0.022		0.021
	**INB**					
		0.8	39	39		39
		-0.8	100	101		101
**Gamma**	**Cost**					
		0.8	0.135	0.137	0.135	0.137
		-0.8	0.133	0.135	0.133	0.135
	**QALYs**					
		0.8	0.021	0.022	0.022	0.021
		-0.8	0.021	0.021	0.021	0.021
	**INB**					
		0.8	95	96	94	96
		-0.8	187	188	186	188
**Inverse Gaussian**	**Cost**					
		0.8	0.155	0.157	0.190	0.157
		-0.8	0.151	0.153	0.192	0.153
	**QALYs**					
		0.8	0.021	0.021	0.023	0.021
		-0.8	0.021	0.021	0.023	0.021
	**INB**					
		0.8	118	119	144	119
		-0.8	201	203	250	203

[a] uBN: unadjusted Bayesian Normal-Normal model;uBGN: unadjusted Bayesian Gamma-Normal models; BFL: Bayesian Full Likelihood

[b] The RMSE corresponding to 2sls is identical to that for 3sls, by definition.

2.1 RMSE details

The RMSE of an estimator $\hat{\theta}$ can be defined as follows

$$\text{RMSE}(\hat{\theta}) = \sqrt{\text{E}((\hat{\theta} - \theta)^2)}$$

where θ is the true value of the parameter.

An equivalent formulation is

$$\text{RMSE}(\hat{\theta}) = \text{Var}(\hat{\theta}) + \text{Bias}(\hat{\theta})^2,$$

with $\text{Var}(\hat{\theta}) = \frac{1}{n_{\text{sims}}} \sum_i \left(\hat{\theta}_i - \bar{\theta}\right)^2$ the empirical variance of the estimator, where $\bar{\theta} = \frac{1}{n_{\text{sims}}} \sum_i \hat{\theta}_i$.

From this, it is clear that since both 2sls and 3sls methods obtain the same point estimate $\hat{\theta}$ for costs, QALYs and INB, this results in the same RMSE, under whichever definition.

On the other hand, the coverage, and the confidence interval width are calculated using the model-based standard errors s_i of the estimators. In the case of the coverage this is

$$\text{Coverage} = \frac{1}{n_{\text{sims}}} \sum_i \mathbb{1} \left(|\hat{\theta}_i - \theta| < z_{\alpha/2} s_i \right),$$

and since the two methods in question obtain different model-based SE for INB (but not for the other two outcomes), these two measures of performance are different between 2sls and 3sls.

References

Congdon, P. (2007), *Bayesian Statistical Modelling, 2nd ed.*, Wiley Series in Probability and Statistics.

Gelman, A. and Hill, J. (2006), *Data Analysis Using Regression and Multilevel/Hierarchical Models*, Analytical Methods for Social Research, Cambridge University Press.

Lunn, D. and Jackson, C. and Best, N. and Thomas, A. and Spiegelhalter, D., (2012) *The BUGS Book: A Practical Introduction to Bayesian Analysis*, Chapman & Hall/CRC Texts in Statistical Science, Taylor & Francis.
